# Variable Selection via Fused Sparse‐Group Lasso Penalized Multi‐state Models Incorporating Molecular Data

**DOI:** 10.1002/bimj.70087

**Published:** 2025-10-27

**Authors:** Kaya Miah, Jelle J. Goeman, Hein Putter, Annette Kopp‐Schneider, Axel Benner

**Affiliations:** ^1^ Division of Biostatistics German Cancer Research Center (DKFZ) Heidelberg Germany; ^2^ Medical Faculty Heidelberg University Heidelberg Germany; ^3^ Department of Biomedical Data Sciences Leiden University Medical Center (LUMC) Leiden The Netherlands; ^4^ Mathematical Institute Leiden University Leiden The Netherlands

**Keywords:** Cox‐type regression, high‐dimensional data, Markov models, regularization, transition‐specific hazards

## Abstract

In multi‐state models based on high‐dimensional data, effective modeling strategies are required to determine an optimal, ideally parsimonious model. In particular, linking covariate effects across transitions is needed to conduct joint variable selection. A useful technique to reduce model complexity is to address homogeneous covariate effects for distinct transitions. We integrate this approach to data‐driven variable selection by extended regularization methods within multi‐state model building. We propose the fused sparse‐group lasso (FSGL) penalized Cox‐type regression in the framework of multi‐state models combining the penalization concepts of pairwise differences of covariate effects along with transition‐wise grouping. For optimization, we adapt the alternating direction method of multipliers (ADMM) algorithm to transition‐specific hazards regression in the multi‐state setting. In a simulation study and application to acute myeloid leukemia (AML) data, we evaluate the algorithm's ability to select a sparse model incorporating relevant transition‐specific effects and similar cross‐transition effects. We investigate settings in which the combined penalty is beneficial compared to global lasso regularization.

**Clinical Trial Registration:** The AMLSG 09‐09 trial is registered with ClinicalTrials.gov (NCT00893399) and has been completed.

## 1 Introduction

**FIGURE 1:**
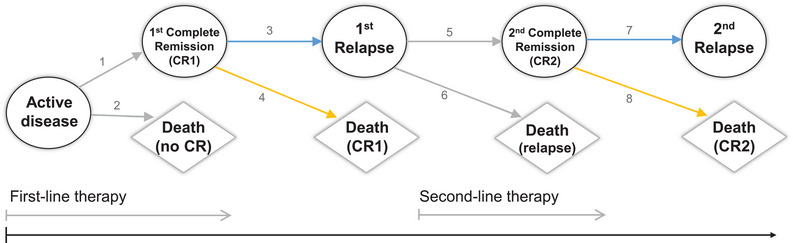
State chart of the multi‐state model for acute myeloid leukemia (AML) with nine states and eight possible transitions represented by arrows. Numbers denote the corresponding transition.

In medical research, prediction models still predominantly make use of composite endpoints such as progression‐ or event‐free survival (EFS). However, these time‐to‐first‐event endpoints do not take into account important aspects of the individual disease pathway and therapy course. Multi‐state models are a natural framework to assess the effect of prognostic factors and treatment on the event history of a patient and to separate risks for the occurrence of distinct events. These extend competing risks analyses of event time endpoints such as time to progression, relapse, remission or death, by modeling the sequence of competing consecutive events on a macro level. In survival analysis, the multi‐state model class is used for event history data where individuals experience a sequence of events over time. Each event is defined by an entry and exit time along with transition types. This paper is motivated by an application to the acute myeloid leukemia (AML) disease pathway. Figure 1 illustrates the event history for AML patients in the form of a state chart of a multi‐state model with nine states and eight transitions. Distinct states are treated as nodes and possible transitions are represented by directed arrows.

To assess how intensities of going from state to state depend on covariates, multi‐state proportional hazards regression models can be used. In the era of precision medicine with increasingly high‐dimensional information on molecular biomarkers, such a holistic analysis of a multi‐state model is of essential interest. For our motivating AML application, we will investigate the effect of biomarkers along with established clinical covariates on the different transitions of the multi‐state model depicted in Figure 1. In particular, incorporating biomarker effects on similar transitions from complete remission (CR) to death or relapse marked by yellow and blue arrows in Figure 1 is of interest. Further, no biomarker effect is expected on, for example, transition 2, i.e., from active disease to early death, since this might rather be related to the initiation of intensive chemotherapy. Thus, effective variable selection strategies for multi‐state models incorporating high‐dimensional molecular data are required to obtain a sparse model and mitigate overfitting. Such data‐driven model building strategies will contribute to a deeper understanding of the individual disease progression and its therapeutic concepts as well as improved personalized prognoses.

This paper focuses on data‐driven variable selection via penalized multi‐state models to capture pathogenic processes and underlying etiologies more accurately. The goal is to select a sparse model based on high‐dimensional molecular data by extended regularization methods. We want to use a priori knowledge about the multi‐state model structure to help simplify it. First, we assume that most biomarkers might have no effect on specific disease transitions, and if they have an effect on one transition, they might have an effect on many. Second, we presume that effect parameters of similar transitions are often of a similar direction and magnitude. Third, biomarker effects might only be relevant for specific transitions. Thus, the parameter space dimensionality should be decreased by setting non‐relevant biomarker effects to zero (i.e., sparsity), identifying similar biomarker effects across distinct transitions (i.e., similarity) and detecting only relevant biomarker effects for specific transitions of interest (i.e., transition‐wise grouping). Furthermore, we want to combine molecular and clinical data by incorporating established clinical predictors into the model that remain unpenalized.

A scoping literature review on model selection methods for multi‐state models was conducted using the PubMed database (http://www.ncbi.nlm.nih.gov/pubmed/advanced). This review identified a variety of approaches addressing model selection in this context. However, to the best of our knowledge, the integration of a priori information regarding structural dependencies between transitions in a multi‐state model remains limited. In the following, we provide a brief overview of current strategies for model selection in multi‐state survival analysis.

Model selection in survival analysis frequently relies on regularization techniques aimed at avoiding to include covariates with negligible effects. In the context of competing risks, Saadati et al. (2018) proposed a lasso‐penalized cause‐specific hazards model for high‐dimensional data, linking separately penalized models by selecting the tuning parameter combination that yields optimal predictive accuracy. Within the multi‐state framework, Sennhenn‐Reulen and Kneib (2016) introduced the structured fusion lasso penalty to enable sparse modeling by considering cross‐transition effects, defined as equal covariate effects across different transitions. Their approach penalizes both the $L_{1}$‐norm of the regression coefficients as well as of all pairwise differences between transitions. Further penalization approaches include the one‐step coordinate descent algorithm for $L_{1}$‐penalization by Dang et al. (2021), and an elastic net‐penalized Markov model by Huang et al. (2018). Beyond penalization, boosting algorithms provide flexible model selection techniques in high‐dimensional settings. Reulen and Kneib (2016) developed a component‐wise functional gradient descent boosting approach for simultaneous variable selection and model specification in multi‐state models, accounting for non‐linear and cross‐transition effects. Complementary work by Edelmann et al. (2020) extended the global test framework to assess equality of regression coefficients across transitions under the Markov assumption. In related efforts to reduce model complexity in terms of dimension reduction, Fiocco et al. (2005, 2008) developed reduced rank regression in proportional hazards models for competing risks and multi‐state data. Alternative frameworks outside the hazard‐based approach also exist, such as direct modeling using accelerated failure time (AFT) models or pseudo‐observations. However, variable selection methods tailored specifically to high‐dimensional multi‐state models within these frameworks have not yet been developed. In this work, we focus on the well‐established Cox‐type framework for multi‐state models, emphasizing hazard‐based model specification. Consequently, alternative direct modeling strategies such as AFT or pseudo‐observation approaches are not pursued further. While the aforementioned approaches provide effective variable selection tools, they often do not fully exploit known structural information inherent to the multi‐state model. For example, general lasso, elastic net, and boosting methods treat transition‐specific effects largely independently or rely on limited assumptions about cross‐transition similarities. Although the structured fusion lasso penalty introduces pairwise constraints on covariate effects across transitions, it does not account for further a priori information on the model structure. Furthermore, flexible variable selection strategies for more complex multi‐state models, that is, moving beyond the illness–death model, have not been sufficiently developed nor implemented, even in low‐dimensional settings. To address these limitations, we propose the fused sparse‐group lasso (FSGL) framework to multi‐state models, which integrates overall sparsity, grouping, and fusion penalties. This approach enables both covariate selection and model structuring informed by a priori knowledge, making it especially suitable for multi‐state models with partially shared effects across transitions or a focus on specific clinically relevant transitions.

In this paper, we introduce the FSGL penalty for multi‐state models combining the concepts of general sparsity, fusion of covariate effects, and transition grouping. To fit such a penalized model, we adapt the alternating direction method of multipliers (ADMM) algorithm to multi‐state Cox‐type hazards regression, leveraging its ability to decompose complex objective functions. Our current implementation focuses on continuous‐time Markov chains with time‐constant covariates and constant baseline hazards.

The remainder of the paper is structured as follows: The methodological background of multi‐state models needed for the proposed adaptation is given in Section 2. Section 3 introduces the FSGL penalty extended to the multi‐state setting. Section 4 describes the general ADMM optimization algorithm for parameter estimation in Subsection 4.1 along with the derived ADMM update steps to fit FSGL penalized multi‐state models in Subsection 4.2. Section 5 shows the results of a proof‐of‐concept simulation study to investigate the regularization performance of the derived algorithm and Section 6 illustrates a real data application to AML patients.

## 2 Methods for Multi‐state Modeling

The following section provides a brief introduction to the multi‐state model class in survival analysis needed for our adapted FSGL penalty to the multi‐state setting. A holistic framework to multi‐state modeling theory can be found in Andersen et al. (1993). Subsection 2.1 introduces the general multi‐state process and defines the concept of transition‐specific Cox proportional hazards regression for multi‐state models. Furthermore, Subsection 2.2 denotes the explicit likelihood formulation in the multi‐state setting along with its derivatives needed for model fitting.

### 2.1 Multi‐state Proportional Hazards Regression Model

Following Andersen and Keiding (2002) and Putter et al. (2007), a multi‐state process is a stochastic process {Z(t),t∈T} with times in $T=\left[0,t_{\max}\right],0<t_{\max}<\infty$, and a finite state space K={1,⋯,K}. The transition probabilities are given as

$$\begin{array}{c} P_{q}\left(s,t\right)=P_{\left[k.k^{\prime }\right]}\left(s,t\right)=P\left(Z\left(t\right)=k^{\prime }\;|\;Z\left(s\right)=k\right) \end{array}$$

for transition $q=[k.k^{\prime }]$ from state k to $k^{\prime },k,k^{\prime }\in K,s,t\in T,s\leq t$, and q∈Q={1,⋯,Q} the set of observable transitions. We assume a continuous‐time Markovian model, that is, the probability for a transition only depends on the current state of the multi‐state process at the current time. The transition intensities are defined as the corresponding derivatives

$$\begin{array}{c} h_{q}\left(t\right)=\lim_{\Delta t↘0}\frac{P_{q}\left(t,t+\Delta t\right)}{\Delta t}. \end{array}$$

To assess the dependence on covariates, these transition‐specific hazard rates can be modeled by separate Cox proportional hazards models for each transition as

$$\begin{array}{c} h_{q}\left(t|x\right)=h_{0,q}\left(t\right)\exp\left\{\beta_{q}^{T}x\right\},\;q=1,\text{…},Q \end{array},$$

for an individual with covariate vector $x={\left(x_{1},\cdots ,x_{P}\right)}^{T}\in R^{P}$, where $h_{0,q}\left(t\right)$ denotes the baseline hazard rate of transition q at time t and $\beta_{q}={\left(\beta_{1,q},\cdots ,\beta_{P,q}\right)}^{T}\in R^{P}$ the vector of transition‐specific regression coefficients. Thus, Cox‐type regression analysis for multi‐state data enables simultaneous modeling of the relationship between covariates and all relevant transitions (Le‐Rademacher et al. 2022).

### 2.2 Multi‐state Likelihood Formulation

In the multi‐state framework, the generalized partial likelihood can be written in terms of a stratified formulation as a product of Cox partial likelihoods for each transition, that is,

$$\begin{array}{c} l\left(\beta \right)=\prod_{q=1}^{Q}l_{q}\left(\beta_{q}\right)=\prod_{q=1}^{Q}\;\prod_{i=1}^{N}{\left(\frac{\exp\{x_{i}^{T}\beta_{q}\}}{\sum_{l\in R_{i,q}}\exp\left\{x_{l}^{T}\beta_{q}\right\}}\right)}^{\delta_{i,q}}, \end{array}$$

where $x_{i}={\left(x_{1;i},\cdots ,x_{P;i}\right)}^{T}\in R^{P}$ denotes the covariate vector of individual i,i=1,⋯,N, $\beta_{q}\in R^{P}$ the transition‐specific regression vector, and $\delta_{i,q}$ the event indicator for transition q (Putter et al. 2007, 2006). $R_{i,q}$ denotes the risk set for individual i with transition q at time $t_{i,q}$. This set includes all individuals who are at risk of experiencing a transition of type q at time $t_{i,q}$. The transition‐specific Cox partial likelihood $l_{q}\left(\beta_{q}\right)$ compares the hazard of the individual with an event at time $t_{i,q}$ to the hazard of all individuals under risk at $t_{i,q}$.

The multi‐state partial likelihood formulation for the stacked regression vector $\beta ={\left(\beta_{1,1},\cdots ,\beta_{1,Q},\beta_{2,1},\cdots ,\beta_{P,Q}\right)}^{T}\in R^{PQ}$ and corresponding extended covariate vector ${\tilde{x}}_{i}={\left(x_{1.1;i},\cdots ,x_{1.Q;i},x_{2.1;i},\cdots ,x_{P.Q;i}\right)}^{T}\in R^{PQ}$, where $x_{p.q;i}$ denotes the observation of covariate $X_{p}$ for transition q of individual i, is then derived as

$$\begin{array}{c} l\left(\beta \right)=\prod_{i=1}^{n}{\left(\frac{\exp\{{\tilde{x}}_{i}^{T}\beta \}}{\sum_{l\in {\tilde{R}}_{i}}\exp\left\{{\tilde{x}}_{l}^{T}\beta \right\}}\right)}^{\delta_{i}}, \end{array}$$

where ${\tilde{R}}_{i}$ denotes the corresponding risk set formulation based on long format data according to de Wreede et al. (2010) with row lines i,j from a total number of n rows of the long format data set. In this format, each individual has a row for each transition for which they are at risk. The negative logarithm of the multi‐state partial likelihood is

(1) $$\begin{array}{c} L\left(\beta \right)=-\log\left[l\left(\beta \right)\right]=\sum_{i=1}^{n}\delta_{i}\left[-{\tilde{x}}_{i}^{T}\beta +\log\left(\sum_{l\in {\tilde{R}}_{i}}\exp\left\{{\tilde{x}}_{l}^{T}\beta \right\}\right)\right]. \end{array}$$

The regression parameters are then estimated by minimizing this negative partial log‐likelihood. The estimate $\hat{\beta }$ is plugged‐in in Breslow's estimate of the cumulative baseline hazard (Putter et al. 2007)

$$\begin{array}{c} {\hat{\Lambda }}_{0,q}\left(t\right)=\sum_{j:t_{j,q}\leq t}\frac{1}{\sum_{l\in R_{j,q}}\exp\left\{x_{l}^{T}{\hat{\beta }}_{q}\right\}}. \end{array}$$

For estimation, we need the first and second derivatives of the Cox partial log‐likelihood function. The score vector is given as

(2) $$\begin{array}{c} U\left(\beta \right)=\frac{\partial }{\partial \beta }\log\left[l\left(\beta \right)\right]=X^{T}\left(\delta -\hat{\mu }\right), \end{array}$$

where $X\in R^{n\times PQ}$ denotes the design matrix, $\delta ={\left(\delta_{1},\cdots ,\delta_{n}\right)}^{T}$ the vector of event indicators and $\hat{\mu }={\left({\hat{\mu }}_{1},\cdots ,{\hat{\mu }}_{n}\right)}^{T}$ the estimated cumulative hazards with elements ${\hat{\mu }}_{i}={\hat{\Lambda }}_{0}\left(t\right)\exp\left\{{\tilde{x}}_{i}^{T}\hat{\beta }\right\}$, and estimated cumulative baseline hazard ${\hat{\Lambda }}_{0}\left(t\right)$ based on long format data. The Hessian matrix is

(3) $$\begin{array}{c} J\left(\beta \right)=\frac{\partial^{2}}{\partial \beta \partial \beta^{T}}\log\left[l\left(\beta \right)\right]=-X^{T}WX \end{array},$$

with $W\in R^{n\times n}$ the weight matrix of the estimated cumulative hazards $\hat{\mu }$ (Goeman 2010; van Houwelingen et al. 2006).

## 3 Fused Sparse‐Group Lasso Penalty

This section describes our adapted fused sparse‐group lasso (FSGL) penalty to multi‐state models as key variable selection strategy for high‐dimensional multi‐state modeling.

For data‐driven model selection, established methods incorporate regularization in the fitting process in order to conduct variable selection (Benner et al. 2010; Heinze et al. 2018). Especially in applications with the number of predictors exceeding numbers of events, regularization is needed in order to obtain a unique and more stable solution of the regression parameters (Salerno and Li 2023). Several regularization methods that perform covariate selection beyond the *least absolute shrinkage and selection operator* (lasso) (Tibshirani 1996) have been developed. These include elastic net (Zou and Hastie 2005), fused lasso (Tibshirani et al. 2005), sparse‐group lasso (Simon et al. 2013), and FSGL (Zhou et al. 2012) penalization. In the multi‐state framework, adapted regularization approaches incorporate the lasso (Saadati et al. 2018; Dang et al. 2021), elastic net (Huang et al. 2018), and structured fusion lasso (Sennhenn‐Reulen and Kneib 2016) for penalized multi‐state modeling. Table 1 gives an overview of existing penalization methods along with their penalty functions as well as their original publications for linear regression models and adaptations to Cox models for survival outcomes.

**TABLE 1:** Examples of penalization methods.

Penalization method	Penalty function	Parameters	Model type
Ridge	${\lambda ∥\beta ∥}_{2}^{2}$	λ>0	Linear (Hoerl and Kennard 1970), Cox (Gray 1992, Verweij and van Houwelingen 1994)
Lasso	${\lambda ∥\beta ∥}_{1}$	λ>0	Linear (Tibshirani 1996), Cox (Tibshirani 1997)
Elastic net	${\alpha ∥\beta ∥}_{1}+\left(1-\alpha \right){∥\beta ∥}_{2}^{2}$	α∈[0,1]	Linear (Zou and Hastie 2005), Cox (Simon et al. 2011)
Fused lasso	$\lambda_{1}\sum_{p=1}^{P}|\beta_{p}|+\lambda_{2}\sum_{p=2}^{P}\left|\beta_{p}-\beta_{p-1}\right|$	$\lambda_{1},\lambda_{2}>0$	Linear (Tibshirani et al. 2005), Cox (Chaturvedi et al. 2014)
Group lasso	$\lambda \sum_{g\in G}\sqrt{p_{g}}{∥\beta_{g}∥}_{2}$	λ>0, groups G, group size $p_{g}$	Linear (Yuan and Lin 2006), Cox (Kim et al. 2012)
Sparse‐group lasso	${\alpha ∥\beta ∥}_{1}+\left(1-\alpha \right)\sum_{g\in G}\sqrt{p_{g}}{∥\beta_{g}∥}_{2}$	α∈[0,1]	Linear & Cox (Simon et al. 2013)
Fused sparse‐group lasso	$\lambda \;[\alpha \gamma ∥\beta {∥}_{1}+\left(1-\gamma \right)∥D\beta {∥}_{1}+\left(1-\alpha \right)\gamma \sum_{g\in G}\sqrt{p_{g}}∥\beta_{g}{∥}_{2}]$	λ>0,α,γ∈[0,1], fusion matrix D	Linear (Beer et al. 2019)
Lasso mstate	$\lambda \sum_{q}\sum_{p}\left|\beta_{p,q}\right|$	λ>0	Competing risks (Saadati et al. 2018), Multi‐state (Dang et al. 2021)
Elastic net mstate	$\left(1-\alpha \right)\sum_{p,q}\beta_{p,q}^{2}+\alpha \sum_{p,q}\left|\beta_{p,q}\right|$	α∈[0,1]	Multi‐state (Huang et al. 2018)
Fusion lasso mstate	$\lambda_{1}\sum_{q}\sum_{p}|\beta_{p,q}|+\lambda_{2}\sum_{q,q^{\prime }}\sum_{p=1}^{P}\left|\beta_{p,q}-\beta_{p,q^{\prime }}\right|$	$\lambda_{1},\lambda_{2}>0$	Multi‐state (Sennhenn‐Reulen and Kneib 2016)

The *fused sparse‐group lasso* penalty, introduced by Zhou et al. (2012) and adapted by Beer et al. (2019) for linear models, provides a combination of lasso, fused, and grouped regularization. Thus, prior information of spatial and group structure can be incorporated into the prediction model. The global lasso penalty fosters overall sparsity. The fusion penalty regularizes absolute pairwise differences of regression coefficients. The group penalty allows variables within the same group to be jointly selected or shrunk to zero.

We propose to transfer this combined penalty to the multi‐state framework based on transition‐specific hazards regression models in order to obtain overall sparsity, link covariate effects across transitions, and incorporate transition grouping. Thus, we advocate the FSGL penalty that provide estimates with three properties:

1. *Sparsity*: The resulting estimator automatically zeros out small estimated coefficients to achieve variable selection and simplify the model (Fan and Li 2002).
2. *Similarity*: The resulting estimator penalizes absolute differences of covariate effects across similar transitions, thus addressing homogeneous cross‐transition effects.
3. *Transition‐wise grouping*: The resulting estimator allows variables within the same transition to be jointly selected or shrunk to zero, thus incorporating transition grouping.

We consider the same set of P (time‐fixed) covariates, for example, biomarkers, for each transition q∈{1,⋯,Q}=Q. Furthermore, we presume a subset S of pairs of similar transitions $\left\{\left(q,q^{\prime }\right):q\neq q^{\prime },q,q^{\prime }\in Q\right\}$, of which we assume that covariate effects across these transitions are of a similar magnitude, i.e., we consider potential cross‐transition effects (Sennhenn‐Reulen and Kneib 2016). The FSGL penalty function is then defined as

(4) $$\begin{array}{cc} p_{\lambda ,\text{FSGL}}\left(\beta \right)=\; & \lambda \left[\alpha \gamma \sum_{q=1}^{Q}\sum_{p=1}^{P}|\beta_{p,q}|+\left(1-\gamma \right)\sum_{(q,q^{\prime })\in S}\sum_{p=1}^{P}\left|\beta_{p,q}-\beta_{p,q^{\prime }}\right|\right.\\ & \;\left.+\;\left(1-\alpha \right)\gamma \sum_{q=1}^{Q}{∥\beta_{q}∥}_{2}\right], \end{array}$$

with transition‐specific regression coefficients $\beta_{p,q}$ of covariate $x_{p},p=1,\cdots ,P$, for transition q, transition‐specific regression vector $\beta_{q}\;\in \;R^{P}$, and tuning parameters λ,α,γ. The tuning parameter λ>0 controls the overall level of regularization, α∈[0,1] balances between global lasso and group lasso and γ∈[0,1] balances between sparse penalties and the fusion penalty (Beer et al. 2019). Thus, the optimal tuning parameter $\lambda_{\text{opt}}$ is chosen at preselected values of α and γ via a model selection criterion (see Section 4.3). For (α,γ)=(1,1), the estimator reduces to the global lasso, for (α,γ)=(0,1) to the group penalty and for (α,γ)=(1,0) or (α,γ)=(0,0) to the fusion penalty. The regression parameter β is estimated by minimizing the penalized negative partial log‐likelihood function, i.e.,

$$\begin{array}{c} \hat{\beta }=\text{arg}\;\min_{\beta }\left[L\left(\beta \right)+p_{\lambda ,\text{FSGL}}\left(\beta \right)\right]. \end{array}$$

## 4 Optimization Algorithm

To effectively fit FSGL‐penalized multi‐state models, it is necessary to employ numerical optimization techniques. Therefore, this section introduces the general concept of the ADMM optimization algorithm in Subsection 4.1 and provides the novel explicitly derived ADMM updating steps specifically tailored for fitting general FSGL penalized multi‐state models in Subsection 4.2. The criterion of selecting optimal penalty parameters is described in Subsection 4.3.

For penalized Cox‐type regression, several numerical optimization algorithms exist for parameter estimation by minimizing the penalized negative likelihood function. Simon et al. (2013) utilize an accelerated generalized gradient algorithm for the sparse‐group lasso penalty. However, the accelerated gradient method depends on the separability of the penalty term across groups of β, so that the fusion penalty can only be applied within groups. For the structured fusion lasso penalty, Sennhenn‐Reulen and Kneib (2016) make use of a penalized iteratively reweighted least squares algorithm. This second‐order optimization has high computation cost and potential convergence problems (Dang et al. 2021). Furthermore, coordinate descent algorithms do not work for the fused lasso penalty due to its non‐separability into a sum of functions of the elements of β that is also not continuously differentiable. Thus, we chose the ADMM optimization algorithm to fit FSGL penalized multi‐state models due to the decomposability of the objective function as well as superior convergence properties.

### 4.1 Alternating Direction Method of Multipliers Algorithm

The *alternating direction method of multipliers* (ADMM) algorithm provides a very general framework for numerical optimization of convex functions. It originates from the 1950s and was developed in the 1970s (Gabay and Mercier 1976; Glowinski and Marroco 1975), but was holistically examined later by Boyd et al. (2011) for a broader conceptuality. The algorithm combines the decomposability of the objective function with superior convergence properties of the method of multipliers (Boyd et al. 2011). Consider the following general optimization problem with respect to a variable $\beta \in R^{P}$

$$\begin{array}{c} \min_{\beta }\;f\left(\beta \right)+g\left(\beta \right), \end{array}$$

where f,g denote convex functions. In the ADMM framework, the generic constrained optimization problem introducing an auxiliary variable $\theta \in R^{P}$ is given as

$$\begin{array}{c} \min_{\beta ,\theta }\;f\left(\beta \right)+g\left(\theta \right)\;\;\;\;\text{subject to}\;\;\theta -\beta =0. \end{array}$$

Thus, the objective function becomes additively separable, which simplifies the subsequent optimization steps. As in the method of multipliers, the augmented Lagrangian function adding an $L_{2}$‐term to enhance optimization stability (Parka and Shin 2022) is given as

$$\begin{array}{cc} & L\left(\beta ,\theta ,\phi \right)=f\left(\beta \right)+g\left(\theta \right)+\phi^{T}\left(\theta -\beta \right)+\frac{\rho }{2}{∥\theta -\beta ∥}_{2}^{2}\\ =\; & L\left(\beta ,\theta ,\nu \right)=f\left(\beta \right)+g\left(\theta \right)+\frac{\rho }{2}{∥\theta -\beta +\nu ∥}_{2}^{2}-\frac{\rho }{2}{∥\nu ∥}_{2}^{2}, \end{array}$$

with Lagrangian multiplier $\phi \in R^{P}$, augmented Lagrangian parameter ρ>0 (i.e., the ADMM step size), and scaled dual variable $\nu =\frac{\phi }{\rho }\in R^{P}$. The general ADMM iterations consist of the following alternating update steps at iteration r+1:

$$\begin{array}{cc} \beta^{r+1} & =\text{arg}\;\min_{\beta }\;L\left(\beta ,\theta^{r},\nu^{r}\right),\\ \theta^{r+1} & =\text{arg}\;\min_{\theta }\;L\left(\beta^{r+1},\theta ,\nu^{r}\right),\\ \nu^{r+1} & =\nu^{r}+\beta^{r+1}-\theta^{r+1}. \end{array}$$

The algorithm comprises a β‐minimization step, a θ‐minimization step, and a dual variable ν‐update. Thus, the usual joint minimization is separated across the decomposition of the objective function over parameters β (e.g., likelihood) and θ (e.g., penalty) into two steps.

As a stopping criterion, Boyd et al. (2010) propose sufficiently small primal and dual residuals, that is,

$$\begin{array}{cc} & ∥\beta^{r+1}-\theta^{r+1}{∥}_{2}<\varepsilon_{1}=\sqrt{P}\varepsilon_{\text{abs}}+\varepsilon_{\text{rel}}\max\{∥\beta^{r}{∥}_{2},∥\theta^{r}{∥}_{2}\}\;\text{and}\\ & ∥\rho \left(\theta^{r+1}-\theta^{r}\right){∥}_{2}<\varepsilon_{2}=\sqrt{P}\varepsilon_{\text{abs}}+\varepsilon_{\text{rel}}∥\nu^{r}{∥}_{2}, \end{array}$$

with absolute and relative tolerances $\varepsilon_{\text{abs}}=10^{-4}$ and $\varepsilon_{\text{rel}}=10^{-2}$.

### 4.2 ADMM for Penalized Multi‐state Models

In the FSGL penalized multi‐state framework, the constrained optimization problem for the stacked regression parameter $\beta \;\in \;R^{PQ}$ is given as

$$\begin{array}{c} \min_{\beta ,\theta }\;f\left(\beta \right)+g\left(\theta \right)\;\;\text{subject to}\;\;\theta_{m}-K_{m}\beta =0,\;m\in \left\{1,\cdots ,M\right\}, \end{array}$$

where f(β)=L(β) is the negative multi‐state partial log‐likelihood function as in (1) and $g\left(\theta \right)=p_{\lambda ,\text{FSGL}}\left(\theta \right)$ is the FSGL penalty function (4) with auxiliary variable $\theta ={\left(\theta_{1},\cdots ,\theta_{M}\right)}^{T}\;\in \;R^{M}$, M=PQ+s+PQ, such that $\theta_{m}=K_{m}\beta$. The penalty structure matrix is defined as $K\;=\;{\left(K_{1}|\cdots |K_{M}\right)}^{T}\;\in \;R^{M\times PQ}$, with elements $k_{ij}\in \left\{-1,0,1\right\}$, such that

$$K_{m}=\left\{\begin{array}{cc} u_{m}, & \text{if}\;m\in \{1,\cdots ,PQ\},\\ d_{m-PQ}, & \text{if}\;m\in \{PQ+1,\cdots ,PQ+s\},\\ G_{m-PQ-s}, & \text{if}\;m\in \{PQ+s+1,\cdots ,PQ+s+Q\}, \end{array}\right.$$

where $u_{m}$ denotes the unit vector of the identity matrix $I_{PQ}\in R^{PQ\times PQ}$ corresponding to the global lasso penalty. The contrast vector $d_{m}$ denotes the (m−PQ)th row vector of the fusion matrix $D\in R^{s\times PQ}$ consisting of s pairs of similar transitions with elements $d_{ij}\;\in \;\left\{-1,1\right\}$ at the corresponding positions of covariates of such similar transitions. These contrast vectors correspond to the fusion penalty, for example, $d_{1}={\left(1,-1,0,\cdots ,0\right)}^{T}$ for the regression coefficients of covariates X1.1 and X1.2 of transitions 1 and 2. $G_{m-PQ-s}\in R^{P\times PQ}$ are the group matrices of the Q transitions consisting of unit vectors, for example, $g_{1}={\left(1,0,\cdots ,0\right)}^{T}$, that indicate the group allocation of a variable to a specific transition, corresponding to the group penalty. For instance, the group matrix for transition 1 of a total of Q=2 transitions and P=2 covariates, such that $\beta ={\left(\beta_{1,1},\beta_{1,2},\beta_{2,1},\beta_{2,2}\right)}^{T}$, is given as

$$\begin{array}{c} G_{1}=\left[\begin{array}{cccc} 1 & 0 & 0 & 0\\ 0 & 0 & 1 & 0 \end{array}\right] \end{array},$$

indicating that the regression coefficients of covariates X1.1 and X2.1 are considered for transition 1 with respect to the group penalty. The penalty structure matrix K is then given as


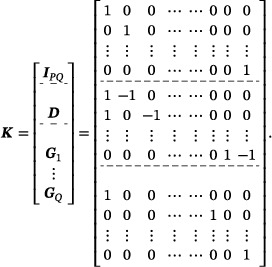

Thus, the total number of rows of the penalty structure matix $K\;\in \;R^{M\times PQ}$ is M=PQ+s+PQ. Optimization of the likelihood and penalty terms are separated and therefore simplified.

For the β‐updating step, Cox estimation of the regression parameter β is performed by numerical algorithms. The gradient descent update is given as $\beta_{\text{GD}}^{r+1}\;=\;\beta^{r}+\varepsilon_{\text{GD}}U\left(\beta^{r}\right)$ using the score vector $U(\beta^{r})$ at iteration r as in (2) and step size $\varepsilon_{\text{GD}}$. The Newton–Raphson update is

$$\beta_{\text{NR}}^{r+1}\;=\;\beta^{r}\;-\;J{\left(\beta^{r}\right)}^{-1}U\left(\beta^{r}\right)$$

using both the gradient $U(\beta^{r})$ and Hessian matrix $J(\beta^{r})$ at iteration r as in (3). The estimation tolerance for the convergence criterion based on the partial log‐likelihood is denoted as ${\text{tol}}_{\text{NR}}$ or ${\text{tol}}_{\text{GD}}$, respectively. A hybrid algorithm as proposed by Goeman (2010) combines adaptive gradient descent and Newton–Raphson to derive β‐estimates in a Cox model. It starts with a single gradient descent step and then switches to Newton–Raphson updating steps. To enable an efficient update of the parameter vector θ, we utilize the proximity operator associated with the $L_{2}$‐norm. Specifically, we employ the vector soft‐thresholding operator defined for $a\in R^{m}$ as

$$S_{\kappa }\left(a\right)={\left(1-\kappa /∥a{∥}_{2}\right)}_{+}a,$$

with $S_{\kappa }\left(0\right)=0$ and ${\left(\cdot \right)}_{+}=\max\left\{0,\cdot \right\}$. This operator performs shrinkage toward zero and yields a closed‐form solution to the proximal mapping of the $L_{2}$‐norm regularizer. As such, it enables a computationally efficient update of θ in each iteration. For a detailed derivation, see Boyd et al. ().

We derive the augmented Lagrangian function along with its first and second derivatives with respect to β as

$$\begin{array}{cc} L(\beta ,\theta ,\nu ) & =f\left(\beta \right)+g\left(\theta \right)+\sum_{m=1}^{M}\left[\nu_{m}\left(\theta_{m}-K_{m}\beta \right)+\frac{\rho }{2}{∥\theta_{m}-K_{m}\beta ∥}_{2}^{2}\right],\\ \frac{\partial }{\partial \beta }L\left(\beta ,\theta ,\nu \right) & =f^{\prime }\left(\beta \right)+\sum_{m=1}^{M}\left[-\nu_{m}K_{m}+\rho \left(-\theta_{m}+K_{m}\beta \right)K_{m}\right]\\ & =U\left(\beta \right)+[\rho \left(\beta^{T}K^{T}-\theta^{T}\right)-\nu^{T}]K\\ & =X^{T}\left(\delta -\hat{\mu }\right)+\left[\rho \left(\beta^{T}K^{T}-\theta^{T}\right)-\nu^{T}\right]K,\\ \frac{\partial }{\partial \beta \partial \beta^{T}}L\left(\beta ,\theta ,\nu \right) & =f^{\prime \prime }\left(\beta \right)+\sum_{m=1}^{M}\left[\rho K_{m}^{T}K_{m}\right]=J\left(\beta \right)+\rho K^{T}K\\ & =-X^{T}WX+\rho K^{T}K, \end{array}$$

with step size ρ>0, scaled dual variable $\nu ={\left(\nu_{1},\cdots ,\nu_{M}\right)}^{T}\in \;R^{M}$, score vector U(β) as in (2), and Hessian matrix J(β) as in (3). Thus, by plugging‐in both derivatives to the Newton–Raphson β‐updating step, our ADMM algorithm for the stacked regression parameter $\beta \;\in \;R^{PQ}$ in a multi‐state model consists of the following steps:

1. Initialize $\beta^{0},\theta^{0}$, and $\nu^{0}$.
2. Update until stopping criterion met:
$$\begin{array}{cc} \beta^{r+1} & =\arg\min_{\beta }L\left(\beta ,\theta^{r},\nu^{r}\right),\\ \theta_{m}^{r+1} & =S_{\frac{\lambda_{m}w_{m}}{\rho }}\left(K_{m}\beta^{r+1}+\nu_{m}^{r}/\rho \right),\;m=1,\cdots ,M,\\ \nu^{r+1} & =\nu^{r}+\rho \left(\theta^{r+1}-K\beta^{r+1}\right), \end{array}$$

where parameter dimensions are $\theta ,\nu \;\in \;R^{M}$. $\lambda_{m}$ denotes the regularization parameters for the global lasso, fusion, and group penalties, respectively, and $w_{m}=\sqrt{P}$ the group weights incorporating the group sizes corresponding to the group penalty. For the stopping criterion, we follow Boyd et al. (2010) adapted for FSGL by Beer et al. (2019) (cf. their Appendix Section 2.2) as

$$\begin{array}{cc} & ∥\theta^{r+1}-K\beta^{r+1}{∥}_{2}<\varepsilon_{1}\;\text{and}\\ & ∥\rho K^{T}\left(\theta^{r+1}-\theta^{r}\right){∥}_{2}<\varepsilon_{2}, \end{array}$$

with $\varepsilon_{1}=\sqrt{PQ}\varepsilon_{\text{abs}}+\varepsilon_{\text{rel}}\max\{∥K\beta^{r+1}{∥}_{2},∥\theta^{r+1}{∥}_{2}\}$, $\varepsilon_{2}=\sqrt{M}\varepsilon_{\text{abs}}+\varepsilon_{\text{rel}}{∥K^{T}\nu^{r+1}∥}_{2}$ and tolerances $\varepsilon_{\text{abs}},\varepsilon_{\text{rel}}$ as in Subsection 4.1. Regarding the ADMM step size ρ>0, we follow Beer et al. (2019) by implementing an adaptive step size proposed by He et al. (2000) to accelerate the convergence of the ADMM algorithm, that is,

$$\begin{array}{c} \rho^{r+1}=\left\{\begin{array}{cc} \tau \rho^{r}, & \text{if}\;∥\theta^{r+1}-K\beta^{r+1}{∥}_{2}>\eta {∥\rho K^{T}\left(\theta^{r+1}-\theta^{r}\right)∥}_{2},\\ \frac{\rho^{r}}{\tau }, & \text{if}\;∥\theta^{r+1}-K\beta^{r+1}{∥}_{2}<\eta {∥\rho K^{T}\left(\theta^{r+1}-\theta^{r}\right)∥}_{2},\\ \rho^{r}, & \text{otherwise}, \end{array}\right. \end{array}$$

where we set τ=2, η=10, and initialize $\rho^{0}=1$. Algorithm 1 provides a summary of the adapted ADMM algorithm to FSGL penalized multi‐state models (*FSGLmstate*).

ALGORITHM 1 **ADMM for fused sparse‐group lasso penalized multi‐state models (*FSGLmstate*).**

**Table:** 

1:	Set $K\in R^{M\times PQ}$, α,γ∈[0,1], $\rho^{0}=1$, and ${\text{tol}}_{\text{GD}}=10^{-6}$ or ${\text{tol}}_{\text{NR}}=10^{-6}$.
2:	**initialize** $\beta^{0}=0_{PQ},\theta^{0}=0_{M},\nu^{0}=0_{M}$.
3:	**repeat**
4:	Update $\beta^{r+1}=\arg\min_{\beta }L\left(\beta ,\theta^{r},\nu^{r}\right)$,
5:	Update $\theta_{m}^{r+1}=S_{\frac{\lambda_{m}w_{m}}{\rho^{r}}}\left(K_{m}\beta^{r+1}+\nu_{m}^{r}/\rho^{r}\right),\;m=1,\text{…},M$,
6:	Update $\nu^{r+1}=\nu^{r}+\rho^{r}\left(\theta^{r+1}-K\beta^{r+1}\right)$,
7:	**until** $∥\theta^{r+1}-K\beta^{r+1}{∥}_{2}<\varepsilon_{1}$ and $∥\rho^{r}K^{T}\left(\theta^{r+1}-\theta^{r}\right){∥}_{2}<\varepsilon_{2}$ for sufficiently small $\varepsilon_{1}$ and $\varepsilon_{2}$.
8:	**obtain** $\hat{\beta }=\hat{\theta }$.

To tackle the dependency of the penalized estimation solution on relative variable scales, standardization is performed for continuous covariates before applying penalization, that is, $x_{p.q}^{*}=\frac{x_{p.q}}{{\hat{\sigma }}_{x_{p.q}}}$, where ${\hat{\sigma }}_{x_{p.q}}$ denotes the empirical standard deviation of $x_{p.q}$. For interpretation, the regression coefficients have to be scaled back after estimation.

The algorithm can be easily amended to situations in which certain covariates should not be penalized (e.g., established clinical predictors). Therefore, we introduce an individual penalty scaling factor $\zeta_{m}\geq 0,m=1,\cdots ,PQ$, which allows different penalties for each variable, that is, $\lambda_{m}=\lambda \zeta_{m}$ (Friedman et al. 2010). Unpenalized parameters get a penalty scaling factor set to zero, that is, $\zeta_{m}=0$ for m∈{1,⋯,PQ}.

Furthermore, it is important to note that the ADMM algorithm does not generate exact zeros for the $\hat{\beta }$‐solution (Andrade et al. 2021; Parka and Shin 2022). However, the estimated auxiliary variable $\hat{\theta }$ is sparse, so that variable selection results are based on the derived estimate $\hat{\theta }$. Thus, we get the final estimated penalized regression vector as $\hat{\beta }=\hat{\theta }$.

### 4.3 Selection of Tuning Parameters

For tuning parameter selection, we focus on the approximate *generalized cross‐validation* (GCV) statistic (Craven and Wahba 1978). This selection criterion was used by Tibshirani et al. (2005) for the fused lasso and Fan and Li (2002) for variable selection in penalized Cox models. GCV is an estimator of the predictive ability of a model (Jansen 2015), which is defined as

$$\begin{array}{c} \text{GCV}\left(\lambda \right)=\frac{L(\hat{\beta })}{N{\left[1-e\left(\lambda \right)/N\right]}^{2}}, \end{array}$$

where λ is a general tuning parameter. The effective number of model parameters for the Cox proportional hazards model in the last step of the Newton–Raphson algorithm iteration (Fan and Li 2002) is approximated as

$$\begin{array}{c} e\left(\lambda \right)=\text{tr}\left[{\left\{\frac{\partial^{2}}{\partial \beta \partial \beta^{T}}L\left(\hat{\beta }\right)+\Sigma_{\lambda }\left(\hat{\beta }\right)\right\}}^{-1}\frac{\partial^{2}}{\partial \beta \partial \beta^{T}}L\left(\hat{\beta }\right)\right], \end{array}$$

with

$$\begin{array}{cc} \Sigma_{\lambda }\left(\hat{\beta }\right) & =\text{diag}\left\{\frac{p^{\prime }\left({\hat{\beta }}_{1,1}\right)}{|{\hat{\beta }}_{1,1}|},\text{…},\frac{p^{\prime }\left({\hat{\beta }}_{P,Q}\right)}{|{\hat{\beta }}_{P,Q}|}\right\} \end{array},$$

and $p^{\prime }\left(\cdot \right)$ denoting the first derivative of the locally quadratic approximated penalty function. The optimal tuning parameter is then selected as $\lambda_{\text{opt}}\;=\;\text{arg}\;\min_{\lambda }\left\{\text{GCV}\left(\lambda \right)\right\}$. For the selection of an optimal combination of multiple tuning parameters, grid search (Tibshirani et al. 2005) along with the Brent optimization algorithm (Brent 1973) is utilized.

## 5 Simulation Study

This section describes the design of a proof‐of‐concept simulation study for evaluating FSGL penalized multi‐state models in terms of variable selection in Subsection 5.1 and illustrates corresponding results in Subsection 5.2. Additional higher‐dimensional simulation results can be found in Appendix A.

### 5.1 Simulation Design

The aim of the following proof‐of‐concept simulation study is to evaluate the variable selection procedure based on FSGL penalized multi‐state models in terms of its ability to select a sparse model distinguishing between relevant transition‐specific effects and equal cross‐transition effects. As a methodological phase II simulation study, it offers empirical evidence to demonstrate validity in finite samples across a limited range of scenarios (Heinze et al. 2024). The corresponding ADEMP criteria (i.e., acronym for *A*im, *D*ata‐generating mechanism, *E*stimand, *M*ethods and *P*erformance measures) according to Morris et al. (2019) of the simulation study are summarized in Table 2. A detailed simulation study plan according to ADEMP‐PreReg (Siepe et al. 2023) can be found in the Supporting Information.

**TABLE 2:** ADEMP criteria of the simulation study according to Morris et al. (2019).

ADEMP criterion	Definition
Aim	Evaluation of sparse variable selection detecting relevant transition‐specific effects and equal cross‐transition effects
Data‐generating mechanism	Multi‐state model based on transition‐specific hazards models
Estimand/target	Regression coefficients
Methods	Unpenalized Cox‐type multi‐state estimation with ADMM optimization;
	Lasso penalized multi‐state model with ADMM optimization (LASSOmstate);
	Fused sparse‐group lasso penalized multi‐state model with ADMM optimization (FSGLmstate)
Performance measures	True positive rate (TPR); False discovery rate (FDR);
	Bias; Mean squared error (MSE)

#### 5.1.1 Data‐Generating Mechanism

In each simulation run, we generate multi‐state data with a sample size of N=1000 from the AML nine‐state model displayed in Figure 1 based on transition‐specific hazards regression as a nested series of competing risks experiments according to Beyersmann et al. (2009). Thus, data have been generated by the following data‐generating process: Waiting times in state l are generated from an exponential distribution with hazards $h_{l\cdot }=\sum_{k=1,k\neq l}^{9}h_{\left[lk\right]}$, l=1,⋯,9. Transition‐specific baseline hazards are set constant to $h_{0,q}\left(t\right)\;=\;0.05$ for all transitions q=1,⋯,8. We synthesize two independent biomarkers as binary covariates $X_{p,i}∼B\left(0.5\right),\;p\;=\;1,2,i\;=\;1,\cdots ,1000$. The true regression parameters for biomarker $X_{1}$ are set to $\beta_{1,1}=1.5$ for transition 1, $\beta_{1,3}=\beta_{1,7}=1.2$ for transitions 3 and 7, $\beta_{1,4}=\beta_{1,8}=-0.8$ for transitions 4 and 8, and $\beta_{1,2}=\beta_{1,5}=\beta_{1,6}=0$ for transitions 2, 5, and 6. Similar transitions are 3 and 7, that is, from first complete remission (CR1) to first relapse and from second complete remission (CR2) to second relapse, as well as 4 and 8, that is, CR1 to death in CR1 and CR2 to death in CR2. Thus, covariate $X_{1}$ has equal effects on these two pairs of similar transitions. Covariate $X_{2}$ has no effect on any transition, that is, $\beta_{2,1}=\cdots =\beta_{2,8}=0$.

#### 5.1.2 Target

Our primary target focuses on the true non‐zero regression coefficients $\beta_{p.q}$ from the penalized multi‐state Cox‐type proportional hazards models

$$\begin{array}{c} h_{q}\left(t|x\right)=h_{0,q}\left(t\right)\exp\left\{\beta_{q}^{T}x\right\},\;q=1,\cdots ,8, \end{array}$$

where $h_{0,q}\left(t\right)$ denotes the baseline hazard rate of transition q at time t, $x={\left(x_{1},\cdots ,x_{P}\right)}^{T}\in R^{P}$ the vector of covariates, and $\beta_{q}\in R^{P}$ the vector of transition‐specific regression coefficients for P covariates, chosen as described in the data‐generating mechanism.

#### 5.1.3 Methods

We aim to compare the *FSGLmstate* algorithm to unpenalized multi‐state Cox‐type estimation and lasso penalized estimation (*LASSOmstate*) based on ADMM optimization. For fitting Cox‐type multi‐state models by ADMM optimization as described in Section 4.2, we chose the following parameter settings: The ADMM variables are initialized as $\beta^{0}=\theta^{0}=\nu^{0}=0$ and the adaptive ADMM step size as $\rho^{0}=1$. The step size in gradient descent is set to $\varepsilon_{\text{GD}}=0.01$, the tolerance of the stopping criterion for Cox estimation ${\text{tol}}_{\text{GD}}\;=\;10^{-6}$, the relative and absolute tolerance for the ADMM stopping criterion $\varepsilon_{\text{rel}}\;=\;10^{-2}$ and $\varepsilon_{\text{abs}}\;=\;10^{-4}$, and the maximum number of iterations to $\max_{\text{iter}}=500$. For each combination of tuning parameters α,γ∈{0,0.25,0.5,0.75,1}, the optimal overall penalty parameter ${\hat{\lambda }}_{\text{opt}}>0$ is selected by minimal GCV over a grid of 20 values of λ∈{0.01,0.023,⋯,362.38,500}, equally spaced on a logarithmic scale.

#### 5.1.4 Performance Measures

Regularization performance is assessed by true positive rates (TPRs) and false discovery rates (FDRs) of variable selection. Median counts of true positives (TPs), true negatives (TNs), false positives (FPs), and false negatives (FNs) of variables over all simulations are calculated. Based on these absolute counts, TPR is calculated as $\text{TPR}=\frac{\text{TP}}{\text{TP+FN}}$. Furthermore, FDR is defined as the number of unrelated variables selected (i.e., FPs) divided by the total number of selected variables, that is, $\text{FDR}=\frac{\text{FP}}{\text{TP+FP}}$.

For quantifying the estimation bias, $\text{Bias}\left(\hat{\beta }\right)=\hat{\beta }-\beta$, for the non‐zero covariates, the mean squared error (MSE) over all simulation iterations is used. The MSE for the non‐zero covariates is defined as

$$\begin{array}{c} {\text{MSE}}_{nz}\left(\hat{\beta }\right)=\frac{1}{d}\sum_{p,q:\beta_{p,q}\neq 0}{\left({\hat{\beta }}_{p,q}-\beta_{p,q}\right)}^{2}, \end{array}$$

where d denotes the number of non‐zero covariates with $\beta_{p,q}\neq 0$ of the true model. The mean bias and mean MSE averaged over the non‐zero predictors over all simulation runs along with Monte Carlo standard errors (MCSEs) are calculated according to Morris et al. (2019).

The number of simulation runs is based on the TPR as one of the primary performance measures of interest. Thus, we need $n_{\text{sim}}\;=\;225$ simulation repetitions per scenario as we aim for TPR ≥0.9 and MCSE(TPR) ≤0.02 and assume $\text{MCSE}(\hat{\text{TPR}})\leq \;0.15$, resulting in $n_{\text{sim}}\;=\;\frac{0.9\cdot 0.1}{0.02^{2}}=225$.

### 5.2 Simulation Results

**FIGURE 2:**
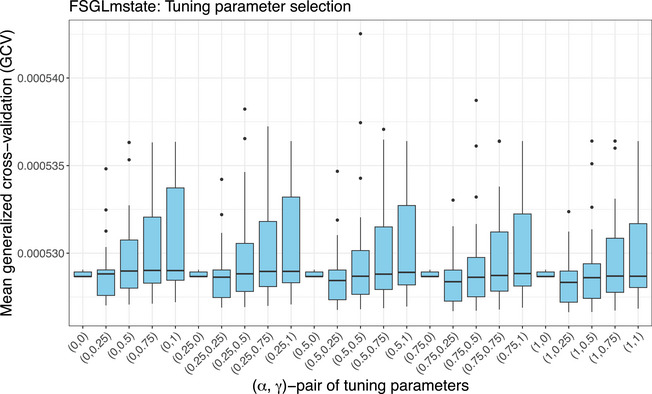
Tuning parameter selection results for FSGLmstate: Mean generalized cross‐validation (GCV) statistics across all preselected combinations of penalty parameters (α,γ) over $n_{\text{sim}}=225$ simulation runs. The pair (α,γ)=(1,1) corresponds to the global lasso penalty.

This section summarizes the main simulation findings, with full results available in the R supplement within the Supporting Information. Tuning parameter selection by minimal GCV for FSGLmstate is illustrated in Figure 2. Boxplots depict mean GCV across combinations of tuning parameter pairs (α,γ) for a grid of penalty parameter λ∈{0.01,⋯,500} over all $n_{\text{sim}}=225$ simulated data sets. For LASSOmstate corresponding to (α,γ)=(1,1), the most frequent lowest GCV is obtained for the optimal tuning parameter ${\hat{\lambda }}_{\text{opt,L}}=8.6$ with mean $\text{GCV}({\hat{\lambda }}_{\text{opt,L}})\cdot 1000=0.52597$ over all simulations. For FSGLmstate, the tuning parameter combination (α,γ)=(1,0.25) yields the most frequent lowest GCV for ${\hat{\lambda }}_{\text{opt,FSGL}}=38.1$ with mean $\text{GCV}({\hat{\lambda }}_{\text{opt,FSGL}})\cdot 1000=0.52663$ over all simulated data sets with the corresponding penalty parameter combination.

**FIGURE 3:**
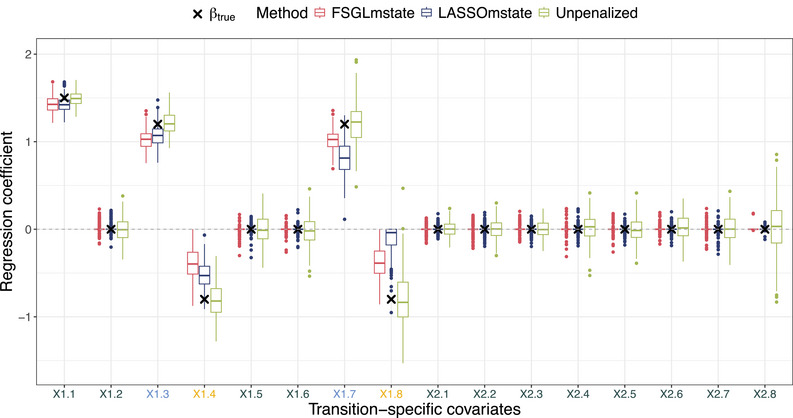
Boxplots of estimated regression coefficients based on simulated data of the nine‐state AML model with eight transitions and two binary covariates. X1.3 and X1.7 as well as X1.4 and X1.8 refer to transitions with true equal effects of covariate $X_{1}$. Covariate $X_{2}$ has no true effect on any transition. Dots depict estimated covariate effects based on ${\hat{\lambda }}_{\text{opt,L}}$ and ${\hat{\lambda }}_{\text{opt,FSGL}}$ of each simulated data set. True underlying covariate effects $\beta_{\text{true}}$ are denoted as crosses (×).

The regularization performance of the FSGLmstate algorithm in comparison to unpenalized and lasso penalized multi‐state Cox‐type estimation is depicted in Figure 3. For our simulation setting with N=1000 observations and PQ=16 regression parameters, unpenalized Cox‐type estimation serves as a gold standard.

The boxplots illustrate the estimated regression coefficients of the binary covariates based on ${\hat{\lambda }}_{\text{opt,L}}$ and ${\hat{\lambda }}_{\text{opt,FSGL}}$. Whereas LASSOmstate identifies the non‐zero effects of $\beta_{1,1}=1.5$, $\beta_{1,3}=\beta_{1,7}=1.2$, and $\beta_{1,4}\;=\;-0.8$, the negative effect of $\beta_{1.8}=-0.8$ for the late transition 8 from CR2 to death in CR2 is set to zero on average. FSGLmstate recognizes the similarity structure of the covariate effect pairs $\beta_{1,3}=\beta_{1,7}=1.2$ as well as $\beta_{1,4}=\beta_{1.8}=-0.8$ while setting all other true negative covariate effects to zero. The unpenalized Cox‐type estimation based on ADMM optimization identifies all non‐zero effects, but inherently does not perform regularization, which results in larger variances for all true negative coefficients.

**FIGURE 4:**
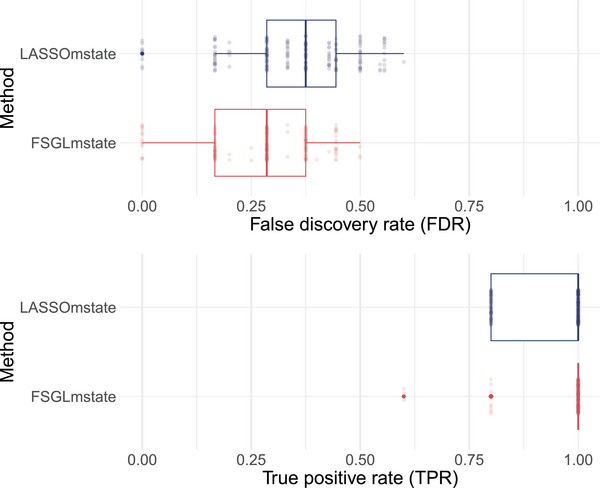
Variable selection results in terms of true positive rates (TPRs) and false discovery rates (FDRs) for LASSOmstate and FSGLmstate. Dots illustrate TPR and FDR of each simulated data set.

Figure 4 depicts variable selection results in terms of TPR and FDR for LASSOmstate and FSGLmstate. Whereas FSGLmstate more often detects all non‐zero regression effects, LASSOmstate's estimated TPR varies between 0.8 and 1.0 (left panel). With regard to FDR, FSGLmstate has a median estimated FDR of 0.29 and LASSOmstate of 0.38 (right panel).

**FIGURE 5:**
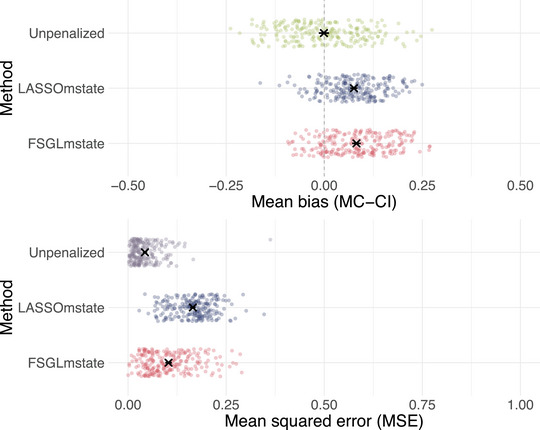
Mean bias and mean squared error (MSE) of estimating the non‐zero covariate effects along with 95% Monte Carlo confidence intervals (MC‐CI). Dots illustrate mean bias and mean MSE of a single simulated data set.

Figure 5 illustrates the mean bias and MSE of estimating the non‐zero covariate effects along with MCSE. As expected, unpenalized Cox‐type estimation exhibits smallest mean bias and MSE of estimating the non‐zero covariate effects in our simulation setting with N=1000 observations. Notably, FSGLmstate provides smaller mean MSEs than LASSOmstate.

## 6 Application to Leukemia Data

The potential of FSGL penalized multi‐state models is further investigated in an illustrative application to leukemia data. The AMLSG 09‐09 study is a randomized phase III trial conducted between 2010 and 2017 at 56 study hospitals in Germany and Austria. The clinical trial evaluated intensive chemotherapy with or without gemtuzumab ozogamicin (GO) in patients with *NPM1*‐mutated AML. Final analysis results for the single and composite endpoints EFS, overall survival (OS), CR rates, and cumulative incidence of relapse (CIR) with long‐term follow‐up are published in Döhner et al. (2023). In conclusion, primary endpoints of the trial in terms of EFS and OS were not met. Additional gene mutation data are available for N=568 study patients.

**FIGURE 6:**
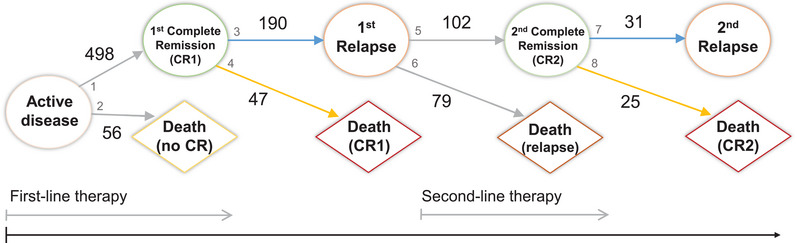
Event counts of the multi‐state model for acute myeloid leukemia (AML) with nine states and eight transitions based on the AMLSG 09‐09 trial data. Gray numbers indicate the corresponding transition.

**FIGURE 7:**
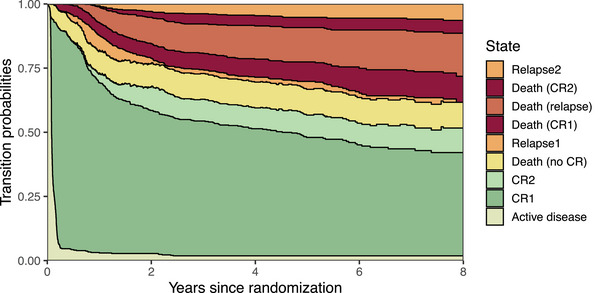
Stacked transition probabilities to all states from randomization derived from the multi‐state model for acute myeloid leukemia (AML) based on the AMLSG 09‐09 trial data. The distance between two adjacent curves represents the probability of being in the corresponding state. CR: Complete remission.

Our motivating nine‐state model for AML along with event counts based on the 09‐09 trial data is illustrated in Figure 6. Late transitions 7 and 8 are rather rarely observed with few events ($E_{7}=31,E_{8}=25$). Derived from this multi‐state model, Figure 7 depicts the stacked transition probabilities to all states from randomization, calculated by the Aalen–Johansen estimator (de Wreede et al. 2011). The probability of being in an intermediate state can fluctuate over time, either increasing or decreasing, while the absorbing state probabilities can only increase over time.

**FIGURE 8:**
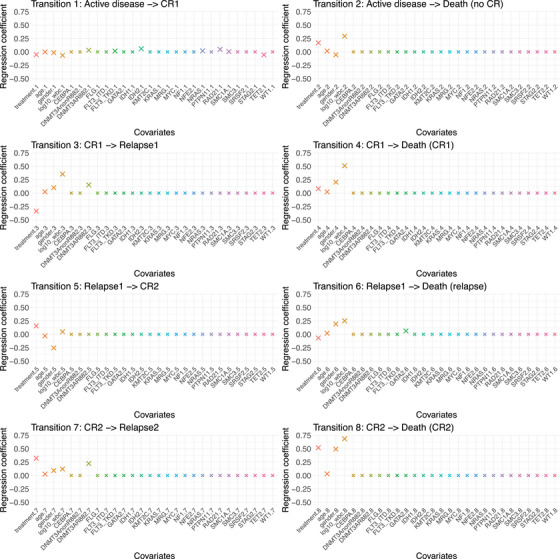
Estimated regression effects of clinical and mutation variables by FSGLmstate separately for each transition derived from the nine‐state model for acute myeloid leukemia (AML) based on the AMLSG 09‐09 trial data. Larger crosses (×) depict non‐zero effects.

For our nine‐state model, we investigate covariate effects of P=24 gene mutations with a prevalence of >3% along with the $P_{c}\;=\;4$ established clinical predictors treatment (GO vs. standard), age (years), sex (male vs. female), and $\log_{10}$ transformed white blood cell count ($10^{9}$ cells/l). Considering these P=28 covariates and Q=8 transitions, we need to incorporate $(P+P_{c})Q\;=\;28\;\cdot \;8\;=\;224$ regression parameters. The clinical predictors should persist unpenalized, thus we apply the FSGL penalty to the remaining 192 mutation parameters. We assume similarity for transitions 3 and 7, that is, CR1 to first relapse and CR2 to second relapse, as well as transitions 4 and 8, that is, CR1 to death in CR1 and CR2 to death in CR2, so that we have s=2 pairs of similar transitions. With respect to a priori expert knowledge on similarity and grouping structures in AML mutations, tuning parameter combinations are investigated for α∈{0.5,0.75,1} with more weight on the global lasso and γ∈{0,0.25,0.5} putting more weight to the fusion penalty. Among all predefined pairs (α,γ), the optimal combination of regularization parameters $\left({\hat{\alpha }}_{\text{opt,FSGL}},{\hat{\gamma }}_{\text{opt,FSGL}}\right)=\left(0.75,0.5\right)$ and ${\hat{\lambda }}_{\text{opt,FSGL}}=20$ is then selected by minimal GCV over the grid λ∈{0.01,⋯,500}. Figure 8 depicts all estimated regression coefficients of clinical and mutation variables by FSGLmstate separately for each transition.

In consistence with final analysis results for CIR, treatment has a negative regression effect on transition 3, that is, from CR1 to first relapse, suggesting an antileukaemic efficacy of intensive chemotherapy including GO (${\hat{\beta }}_{\text{treatment.3}}=-0.34$). With respect to molecular markers, mutations of the DNA methylation gene *DNMT3A* ^R882^ are selected for transition 3 from CR1 to first relapse, as well as for transition 7 from CR2 to second relapse. This result aligns with accompanying gene mutation analyses of Cocciardi et al. (2025), where *DNMT3A* ^R882^ mutations were associated with an increased CIR.

## 7 Discussion

In this paper, we propose FSGL penalized multi‐state models for data‐driven variable selection and dimension reduction in order to capture pathogenic disease processes more accurately while incorporating clinical and molecular data. The objective was to select a sparse model based on high‐dimensional molecular data by extended regularization methods. We adapted the ADMM algorithm to FSGL penalized multi‐state models combining the penalization concepts of general sparsity, pairwise differences of covariate effects along with transition grouping. Thus, FSGL penalized multi‐state models tackle sparse model building while incorporating a priori information about the covariate and transition structure into a prediction model. Furthermore, the ADMM algorithm can quite efficiently handle large‐scale problems due to the decomposability of the objective function as well as superior convergence properties.

The proof‐of‐concept simulation study evaluated the FSGLmstate algorithm's regularization performance to select a sparse model incorporating only relevant transition‐specific effects and similar cross‐transition effects. Compared to unpenalized and global lasso penalized estimation, FSGLmstate identifies similarity and grouping structures depending on the choices of the corresponding tuning parameters. Since the number of parameters in multi‐state models is generally large even when there are few covariates, our method requires a moderate to large sample size for effective estimation.

The data application on a phase III AML trial illustrated the utility of an FSGL penalized multi‐state model to reduce model complexity while combining clinical and molecular data. Whereas an unpenalized nine‐state model incorporating all established clinical predictors along with high‐dimensional mutation information based on the study data suffers from overfitting due to few events per variable, our FSGLmstate approach allows to fit a penalized nine‐state model combining clinical predictors and mutation variables.

Several improvements and extensions of the proposed FSGL penalty to multi‐state models offer further research directions. One limitation of our work is that time‐dependent covariates, for example, allogeneic stem cell transplantation, and time‐dependent effects are not yet incorporated. Beyond the current scope, future work could explore distributional baseline hazards. Furthermore, post‐selection inference requires to be investigated. Besides, the algorithm needs further adaptations to enhance computational speed and efficiently handle very high dimensions with P≫N. To this end, our implementation is computationally intensive given the multiple tuning parameter setting as well as the double optimization scheme. Thus, high‐dimensional simulation studies were limited by computation times and cloud computing resources. Furthermore, different tuning parameter selection criteria should be investigated and extensive phase III simulations for empirical method comparisons are required to evaluate the performance of our variable selection method across a wide range of settings.

## Ethics Statement

The DFG project *Mehrstadienmodellierung zur Prüfung prognostischer und prädiktiver Biomarker in der akuten myeloischen Leukämie* (grant no. 514653984) was approved by the Ethics Committee of Ulm University (application no. 249/21). The AMLSG 09‐09 clinical trial was approved by the ethics committees at all sites.

## Consent

Written informed consent was obtained from all patients involved in the study.

## Conflicts of Interest

The authors declare no conflicts of interest.

## Open Research Badges

This article has earned an Open Data badge for making publicly available the digitally‐shareable data necessary to reproduce the reported results. The data is available in the Supporting Information section.

This article has earned an open data badge “**Reproducible Research**” for making publicly available the code necessary to reproduce the reported results. The results reported in this article could fully be reproduced.

## Supporting information

Supporting Information

## Figures and Tables

**FIGURE A1:**
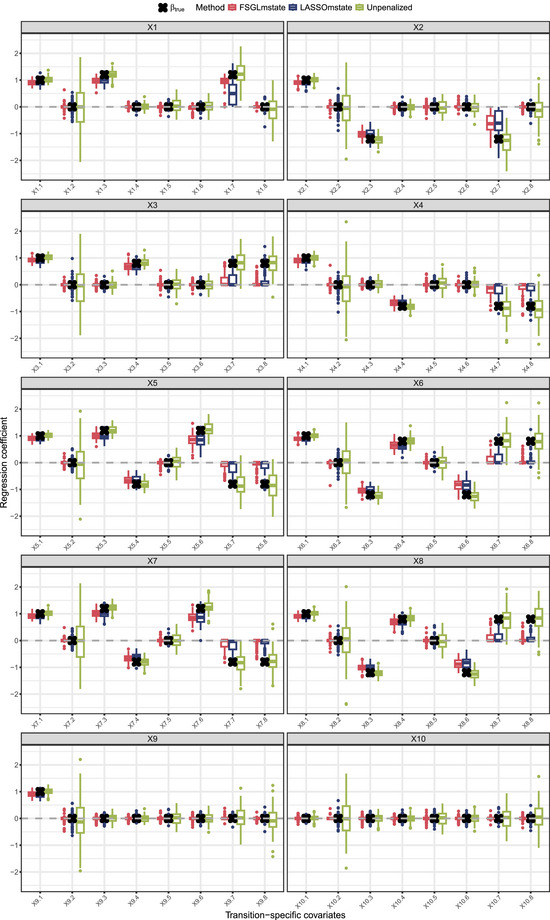
Scenario B: Boxplots of estimated regression coefficients based on simulated data of the nine‐state AML model with eight transitions and P=10 binary covariates. Dots depict estimated covariate effects based on ${\hat{\lambda }}_{\text{opt,L}}$ and ${\hat{\lambda }}_{\text{opt,FSGL}}$ of each simulated data set. True underlying covariate effects $\beta_{\text{true}}$ are denoted as crosses (×).

**FIGURE A2:**
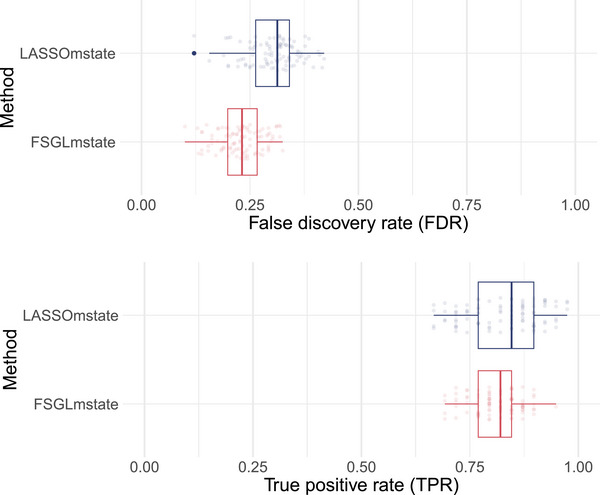
Scenario B: Variable selection results in terms of true positive rates (TPR) and false discovery rates (FDR) for LASSOmstate and FSGLmstate. Dots illustrate TPR and FDR of each simulated data set.

**FIGURE A3:**
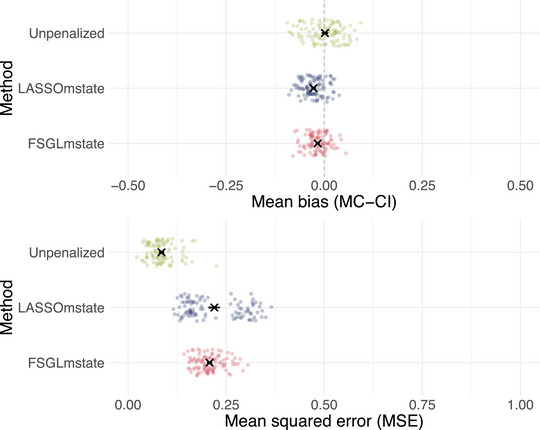
Scenario B: Mean bias and mean squared error (MSE) of estimating the non‐zero covariate effects along with 95% Monte Carlo confidence intervals (MC‐CI). Dots illustrate mean bias and mean MSE of each simulated data set.

**FIGURE A4:**
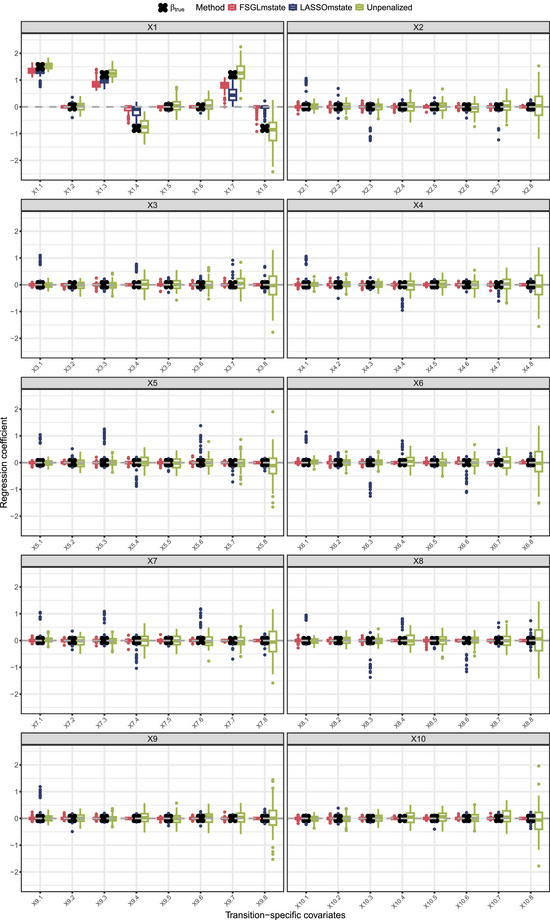
Scenario C: Boxplots of estimated regression coefficients based on simulated data of the nine‐state AML model with eight transitions and P=10 binary covariates. Dots depict estimated covariate effects based on ${\hat{\lambda }}_{\text{opt,L}}$ and ${\hat{\lambda }}_{\text{opt,FSGL}}$ of each simulated data set. True underlying covariate effects $\beta_{\text{true}}$ are denoted as crosses (×).

**FIGURE A5:**
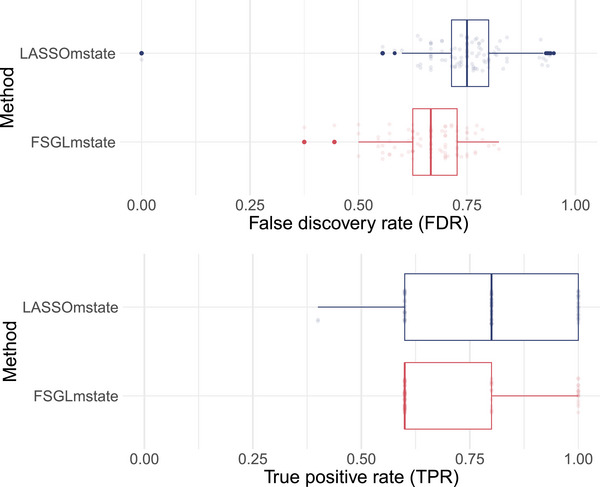
Scenario C: Variable selection results in terms of true positive rates (TPR) and false discovery rates (FDR) for LASSOmstate and FSGLmstate. Dots illustrate TPR and FDR of each simulated data set.

**FIGURE A6:**
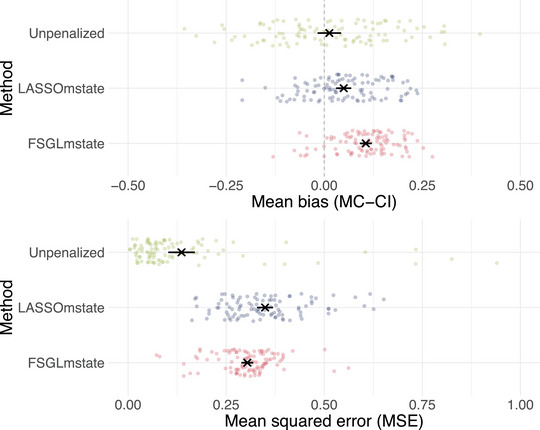
Scenario C: Mean bias and mean squared error (MSE) of estimating the non‐zero covariate effects along with 95% Monte Carlo confidence intervals (MC‐CI). Dots illustrate mean bias and mean MSE of each simulated data set.

## Data Availability

Due to legal restrictions, data of the AMLSG 09‐09 clinical trial are not publicly available. All implementations and statistical analyses are performed utilizing the statistical computing language R, version 4.4.1 (R Core Team 2024), along with the R packages mstate (de Wreede et al. 2011), penalized (Goeman 2010), and penMSM (Reulen 2015), among others. R code to reproduce simulation study results and manuscript figures is available in the Supporting Information along with the GitHub R package FSGLmstate (https://github.com/k‐miah/FSGLmstate).

## Appendix A Simulation Study: High‐Dimensional Results

This section provides further simulation results in addition to the findings reported in Section 5.

### A.1 Simulation Design

The overall simulation design remains as described in Subsection 5.1, with the addition of two scenarios B and C, which include additional covariates in terms of high‐dimensional data, further regression parameter settings and sample size detailed below.

#### A.1.1 Data‐Generating Mechanism

In each simulation run ($n_{\text{sim}}=100$), we generate multi‐state data from the acute myeloid leukemia (AML) multi‐state model displayed in Figure 1 based on transition‐specific hazards regression as a nested series of competing risks experiments according to Beyersmann et al. (2009). Thus, data have been generated by the following data‐generating processes:

**Scenario B:**

- Sample size N=500,
- P=10 binary covariates $X_{p,i}\;∼\;B\left(0.5\right),\;p\;=\;1,\cdots ,10,i\;=\;1,\cdots ,500$,
- Constant transition‐specific baseline hazards $h_{0,q}\left(t\right)=0.05,q=1,\cdots ,8$,
- Waiting times $T_{l}∼\text{Exp}\left(h_{l\cdot }\right)$ in state l with hazard $h_{l\cdot }=\sum_{k=1,k\neq l}^{9}h_{\left[lk\right]}$, l=1,⋯,9,
- True regression parameters:
  $\beta_{1,\cdot }={\left(1,0,1.2,0,0,0,1.2,0\right)}^{T}$,
  $\beta_{2,\cdot }={\left(1,0,-1.2,0,0,0,-1.2,0\right)}^{T}$,
  $\beta_{3,\cdot }={\left(1,0,0,0.8,0,0,0.8,0.8\right)}^{T}$,
  $\beta_{4,\cdot }={\left(1,0,0,-0.8,0,0,-0.8,-0.8\right)}^{T}$,
  $\beta_{5,\cdot }={\left(1,0,1.2,-0.8,0,1.2,-0.8,-0.8\right)}^{T}$,
  $\beta_{6,\cdot }={\left(1,0,-1.2,0.8,0,-1.2,0.8,0.8\right)}^{T}$,
  $\beta_{7,\cdot }={\left(1,0,1.2,-0.8,0,1.2,-0.8,-0.8\right)}^{T}$,
  $\beta_{8,\cdot }={\left(1,0,-1.2,0.8,0,-1.2,0.8,0.8\right)}^{T}$,
  $\beta_{9,\cdot }={\left(1,0,0,0,0,0,0,0\right)}^{T}$,
  $\beta_{10,\cdot }={\left(0,0,0,0,0,0,0,0\right)}^{T}$.

**Scenario C**:

- Sample size N=500,
- P=10 binary covariates $X_{p,i}\;∼\;B\left(0.5\right),\;p\;=\;1,\cdots ,10,i\;=\;1,\cdots ,500$,
- Constant transition‐specific baseline hazards $h_{0,q}\left(t\right)=0.05,q=1,\cdots ,8$,
- Waiting times $T_{l}∼\text{Exp}\left(h_{l\cdot }\right)$ in state l with hazard $h_{l\cdot }=\sum_{k=1,k\neq l}^{9}h_{\left[lk\right]}$, l=1,⋯,9,
- True regression parameters:
  $\beta_{1,\cdot }={\left(1.5,0,1.2,-0.8,0,0,1.2,-0.8\right)}^{T}$,
  $\beta_{2,\cdot }=\cdots =\beta_{10,\cdot }={\left(0,0,0,0,0,0,0,0\right)}^{T}$.

The number of simulation runs is determined based on the target true positive rate (TPR). To achieve a TPR of at least 0.9 with a Monte Carlo standard error (MCSE) of 0.03 or less, we require $n_{\text{sim}}=\frac{0.9\cdot 0.1}{0.03^{2}}=100$ simulation repetitions per scenario.

#### A.1.2 Methods

We aim to compare the *FSGLmstate* algorithm to unpenalized multi‐state Cox‐type estimation and lasso penalized estimation (*LASSOmstate*) based on alternating direction method of multipliers (ADMM) optimization. For fitting Cox‐type multi‐state models by ADMM optimization as described in Section 4.2, we chose the following parameter settings:

- Augmented Lagrangian parameter $\rho^{0}=1$,
- Step size in gradient descent $\varepsilon_{\text{GD}}=0.01$,
- Tolerance of stopping criterion for Cox estimation ${\text{tol}}_{\text{GD}}=10^{-6}$,
- Relative/absolute tolerance for ADMM stopping criterion $\varepsilon_{\text{rel}}=10^{-2},\varepsilon_{\text{abs}}=10^{-4}$,
- Maximum number of iterations $\max_{\text{iter}}=500$.

For each combination of tuning parameters α,γ∈{0,0.25,0.5,0.75,1}, the optimal overall penalty parameter ${\hat{\lambda }}_{\text{opt}}>0$ is selected by minimal generalized cross‐validation (GCV) over a grid of 20 values of λ∈{0.01,0.023,⋯,362.38,500}, equally spaced on a logarithmic scale.

### A.2 Simulation Results

This section illustrates the additional simulation findings, with full results available in the R supplement within the Supporting Information.

#### A.2.1 Scenario B: P=10 Covariates (Non‐sparse)

Figure A1 depicts the regularization performance of the FSGLmstate algorithm compared to unpenalized and lasso‐penalized multi‐state Cox‐type estimators. Figure A2 shows variable selection results in terms of TPR and false discovery rate (FDR) for LASSOmstate and FSGLmstate. Figure A3 illustrates the mean bias and mean squared error (MSE) of estimating the non‐zero covariate effects along with MCSE. Consequently, in terms of model selection consistency, the proof‐of‐concept simulation study reported in Section 5 based on sample sizes with N=1000 provides higher TPR as well as lower FDR compared to the additional simulation findings based on N=500.

#### A.2.2 Scenario C: P=10 Covariates (Sparse)

Figure A4 depicts the regularization performance of the FSGLmstate algorithm compared to unpenalized and lasso‐penalized multi‐state Cox‐type estimators. Figure A5 presents variable selection performance of LASSOmstate and FSGLmstate in terms of TPR and FDR. Figure A6 displays the mean bias and MSE of estimating the non‐zero covariate effects, along with the corresponding MCSE. Consequently, in terms of model selection consistency, the proof‐of‐concept simulation study reported in Section 5 based on sample sizes with N=1000 yields higher TPR as well as lower FDR than the additional simulation findings based on N=500.
