# Supplementary figures and images for: Variable Selection via Fused Sparse‐Group Lasso Penalized Multi‐state Models Incorporating Molecular Data
Source: Biom J. 2025 Oct 27;67(6):e70087. doi: 10.1002/bimj.70087 (PMC12559784; doi:10.1002/bimj.70087)

# LASSOmstate: Tuning parameter selection

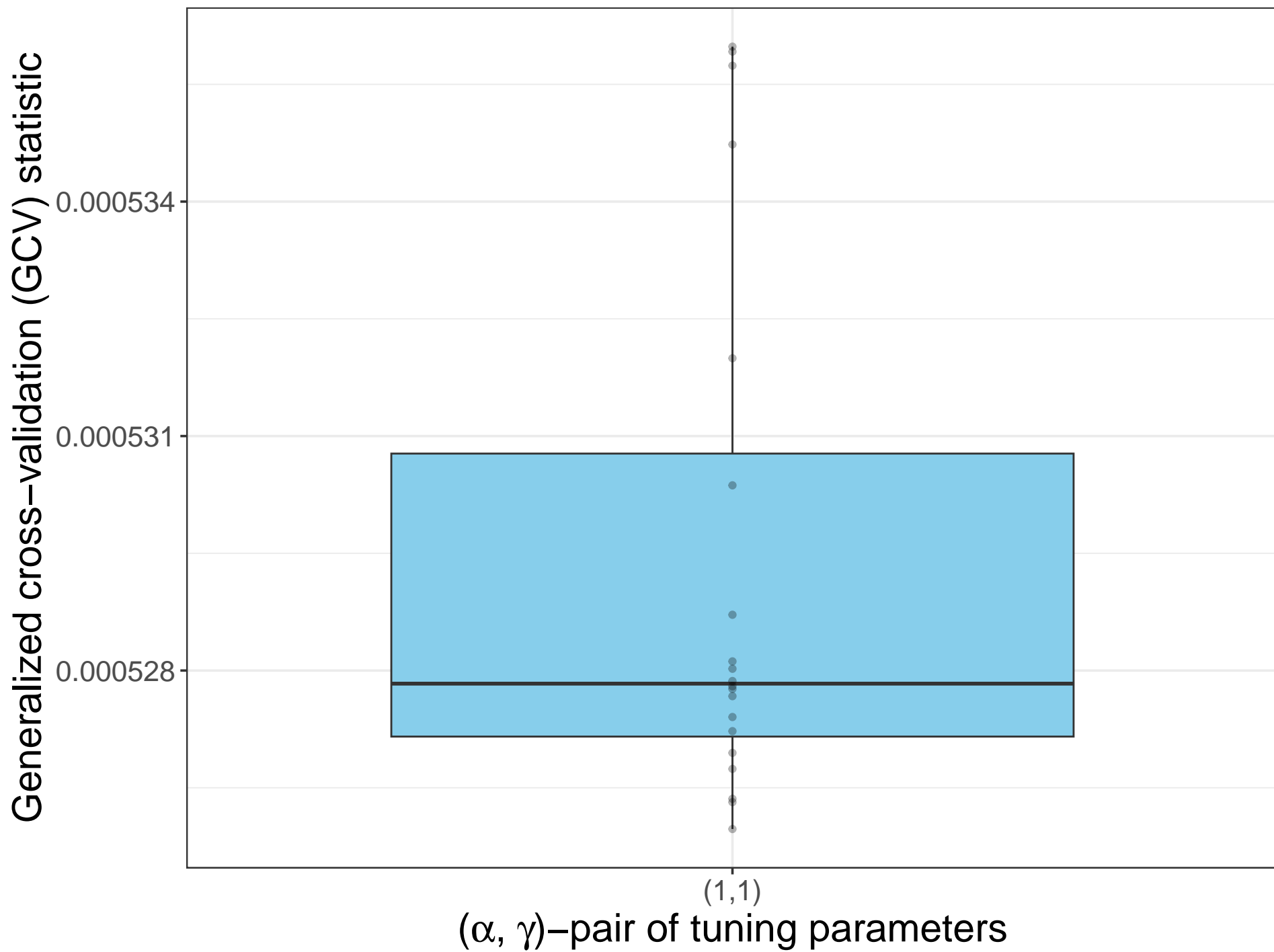

Supplement: Supplementary file 1 — Supporting Information [file BIMJ-67-e70087-s001.zip › bimj2025-sup-code/figures/Boxplot_GCV_LASSOmstate_nobs1000.pdf]

×  $\beta_{\text{true}}$  Method ▢ FSGLmstate ▢ LASSOmstate ▢ Unpenalized

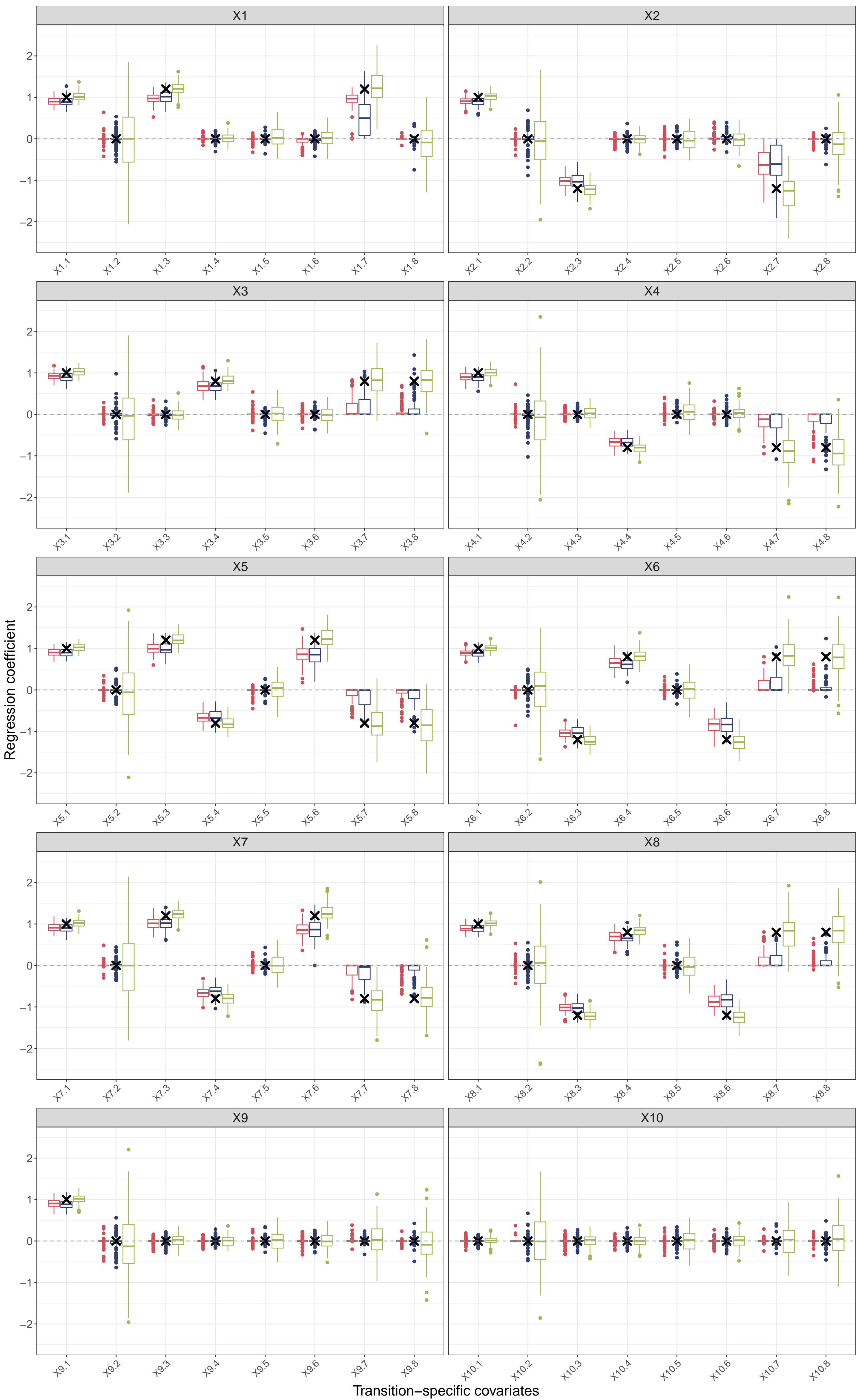

Supplement: Supplementary file 1 — Supporting Information [file BIMJ-67-e70087-s001.zip › bimj2025-sup-code/figures/Figure_A1_Boxplots_coef_P10_nsim100_nobs500.pdf]

Method

LASSOmstate

FSGLmstate

0.00

0.25

0.50

0.75

1.00

False discovery rate (FDR)

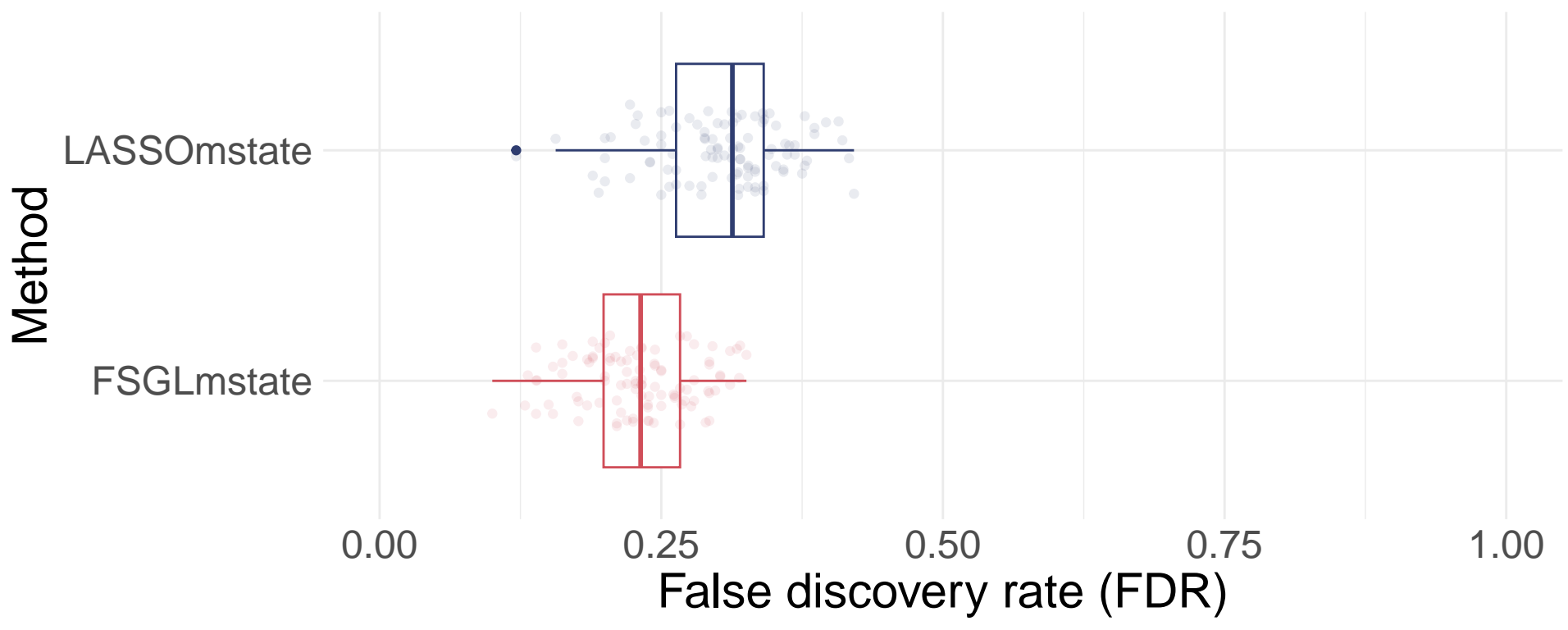

Supplement: Supplementary file 1 — Supporting Information [file BIMJ-67-e70087-s001.zip › bimj2025-sup-code/figures/Figure_A2_FDR_nsim100_nobs500.pdf]

Method

LASSOmstate

FSGLmstate

0.00

0.25

0.50

0.75

1.00

True positive rate (TPR)

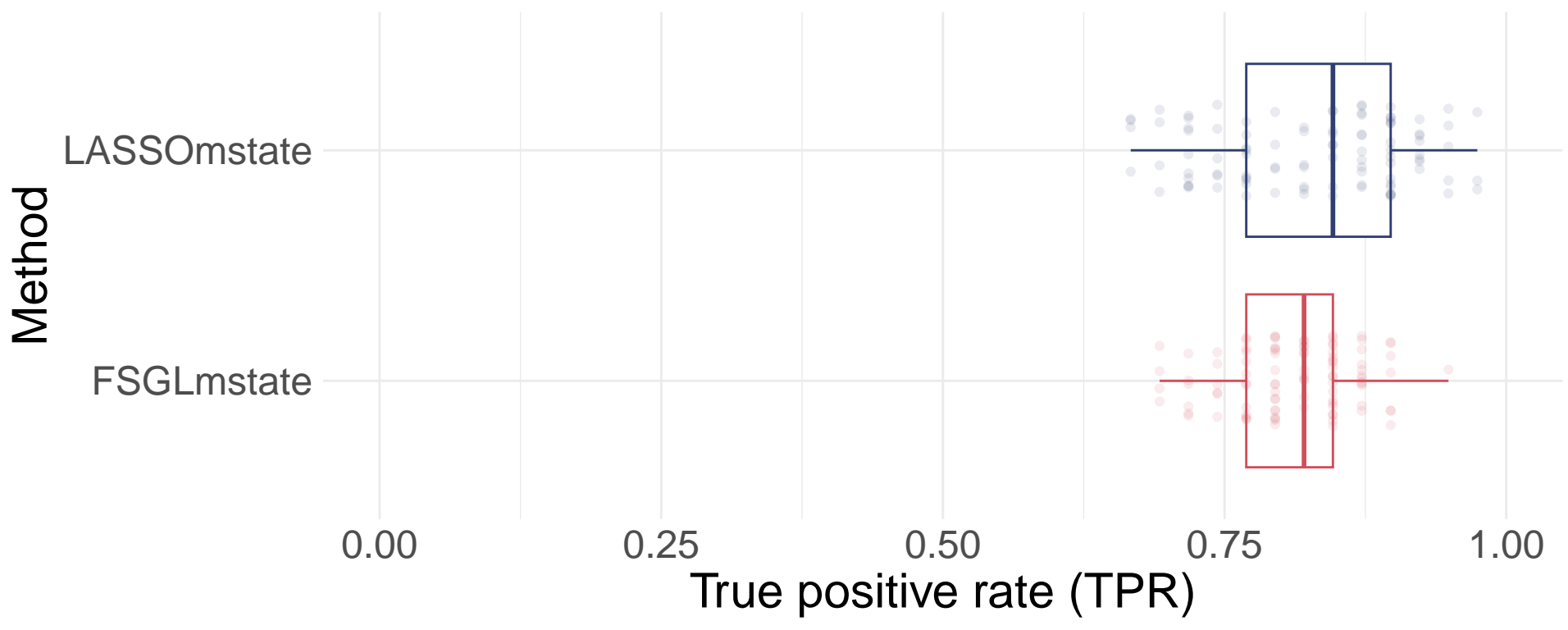

Supplement: Supplementary file 1 — Supporting Information [file BIMJ-67-e70087-s001.zip › bimj2025-sup-code/figures/Figure_A2_TPR_nsim100_nobs500.pdf]

Method

Unpenalized

LASSOmstate

FSGLmstate

-0.50

-0.25

0.00

0.25

0.50

Mean bias (MC-CI)

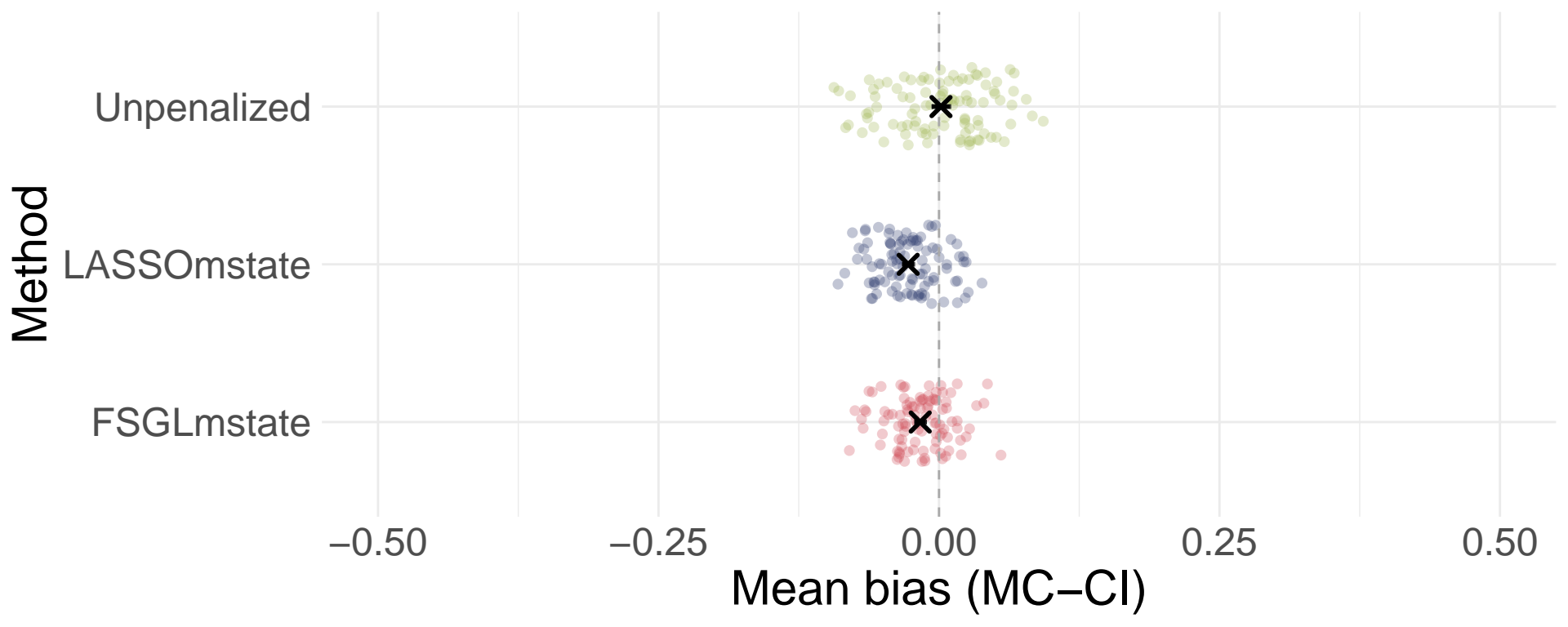

Supplement: Supplementary file 1 — Supporting Information [file BIMJ-67-e70087-s001.zip › bimj2025-sup-code/figures/Figure_A3_mean-bias_nsim100_nobs500.pdf]

Method

Unpenalized

LASSOmstate

FSGLmstate

0.00

0.25

0.50

0.75

1.00

Mean squared error (MSE)

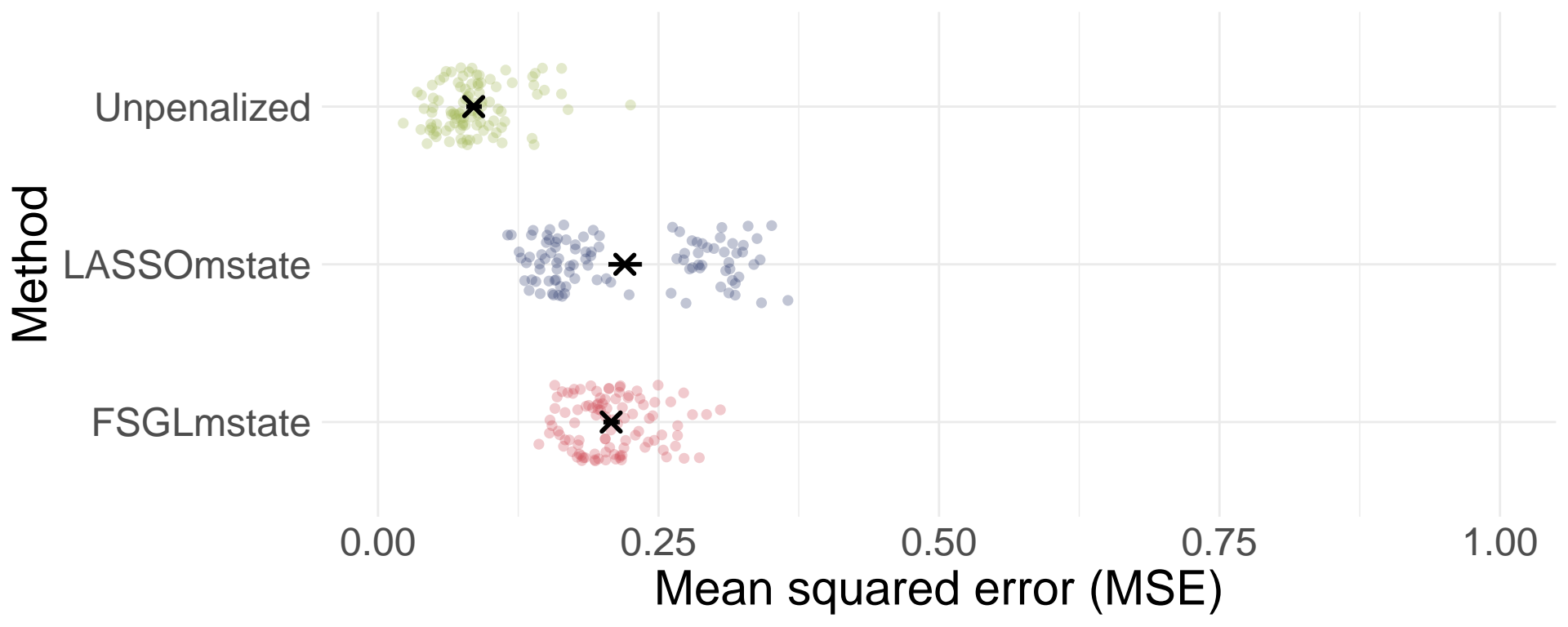

Supplement: Supplementary file 1 — Supporting Information [file BIMJ-67-e70087-s001.zip › bimj2025-sup-code/figures/Figure_A3_MSE_nsim100_nobs500.pdf]

$\times \beta_{\text{true}}$  Method ■ FSGLmstate ■ LASSOmstate ■ Unpenalized

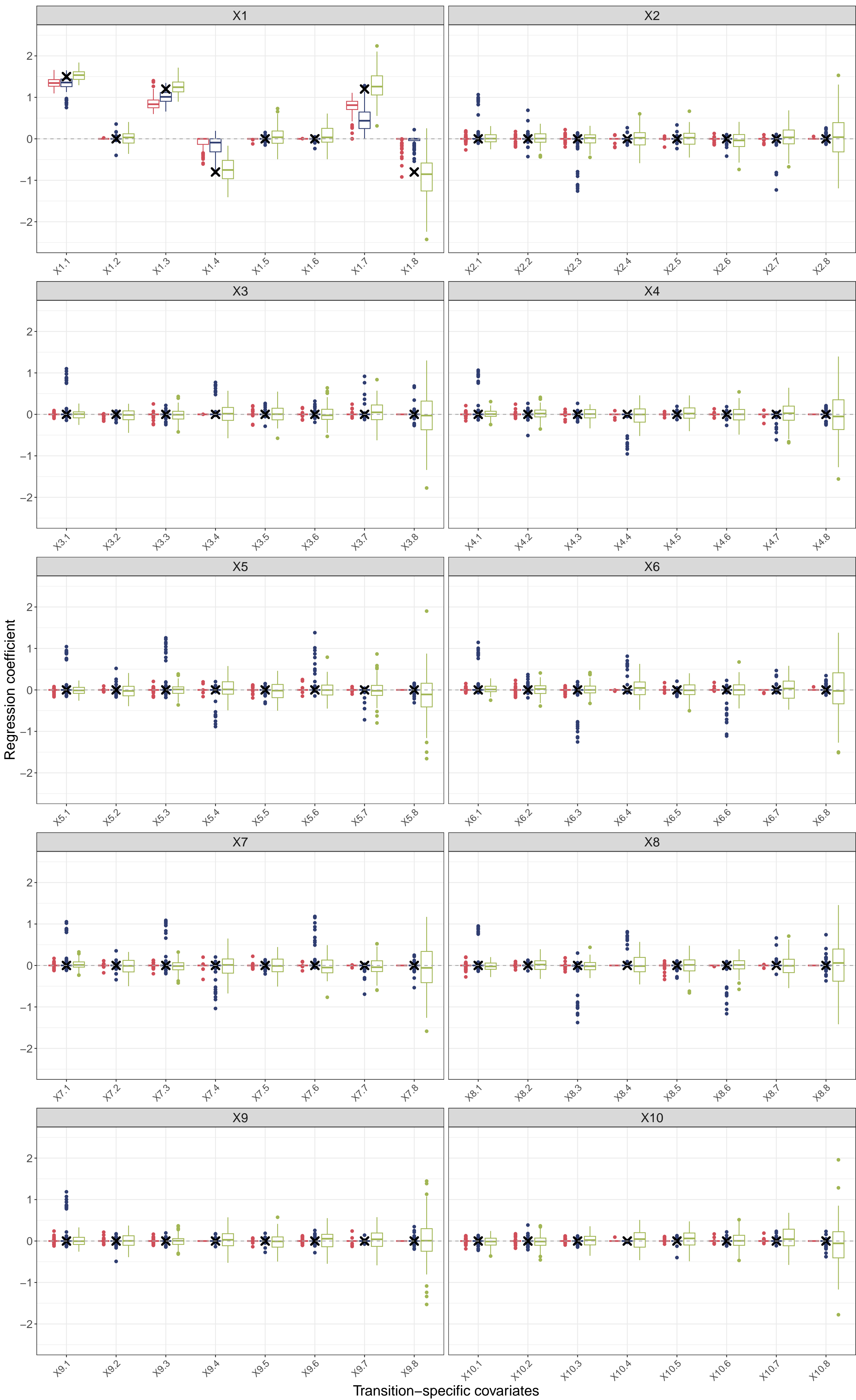

Supplement: Supplementary file 1 — Supporting Information [file BIMJ-67-e70087-s001.zip › bimj2025-sup-code/figures/Figure_A4_Boxplots_coef_P10_nsim100_nobs500.pdf]

Method

LASSOmstate

FSGLmstate

0.00

0.25

0.50

0.75

1.00

False discovery rate (FDR)

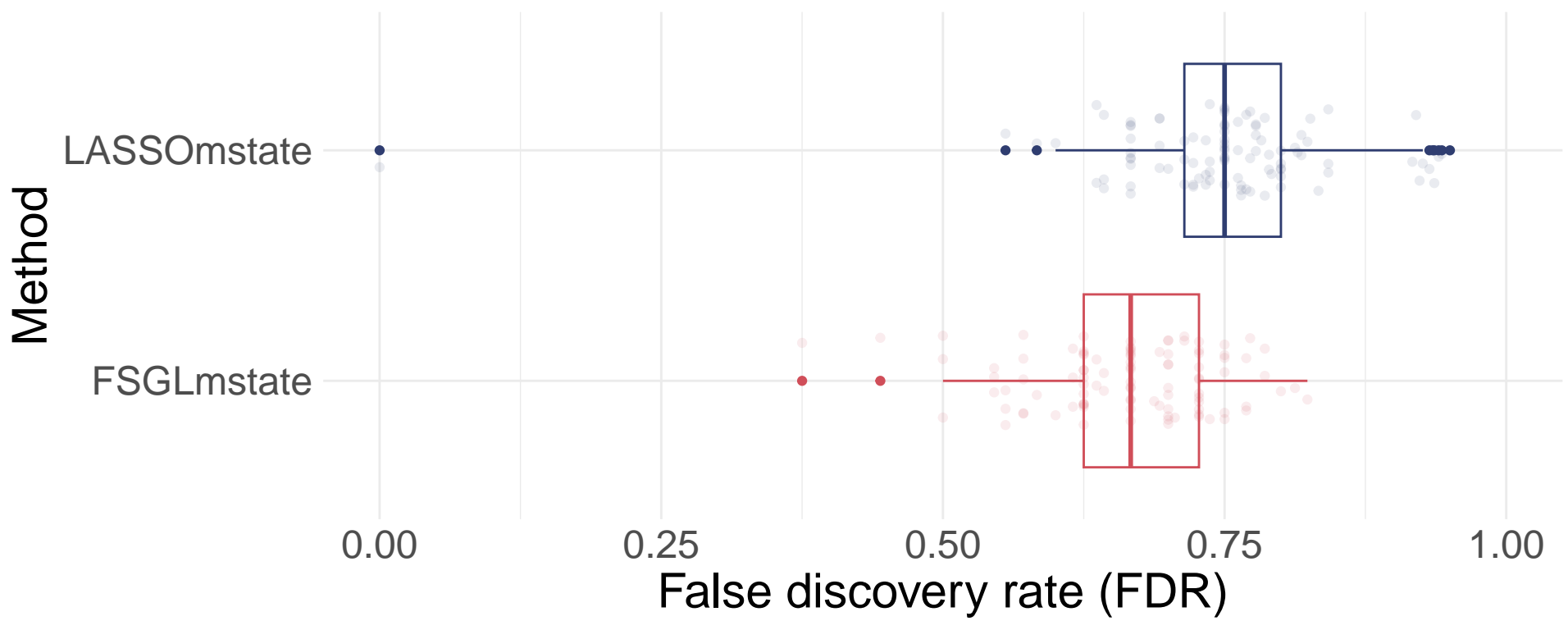

Supplement: Supplementary file 1 — Supporting Information [file BIMJ-67-e70087-s001.zip › bimj2025-sup-code/figures/Figure_A5_FDR_nsim100_nobs500.pdf]

Method

LASSOmstate

FSGLmstate

0.00

0.25

0.50

0.75

1.00

True positive rate (TPR)

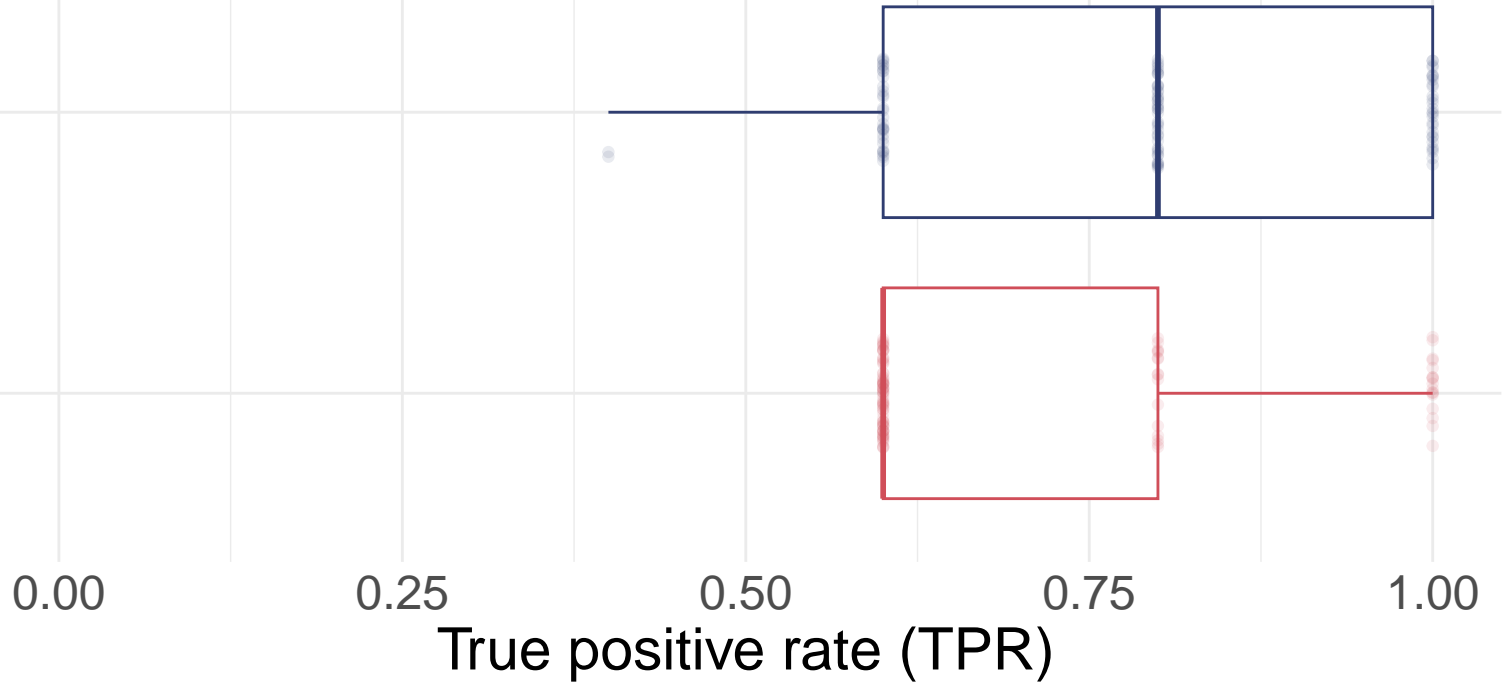

Supplement: Supplementary file 1 — Supporting Information [file BIMJ-67-e70087-s001.zip › bimj2025-sup-code/figures/Figure_A5_TPR_nsim100_nobs500.pdf]

Method

Unpenalized

LASSOmstate

FSGLmstate

-0.50

-0.25

0.00

0.25

0.50

Mean bias (MC-CI)

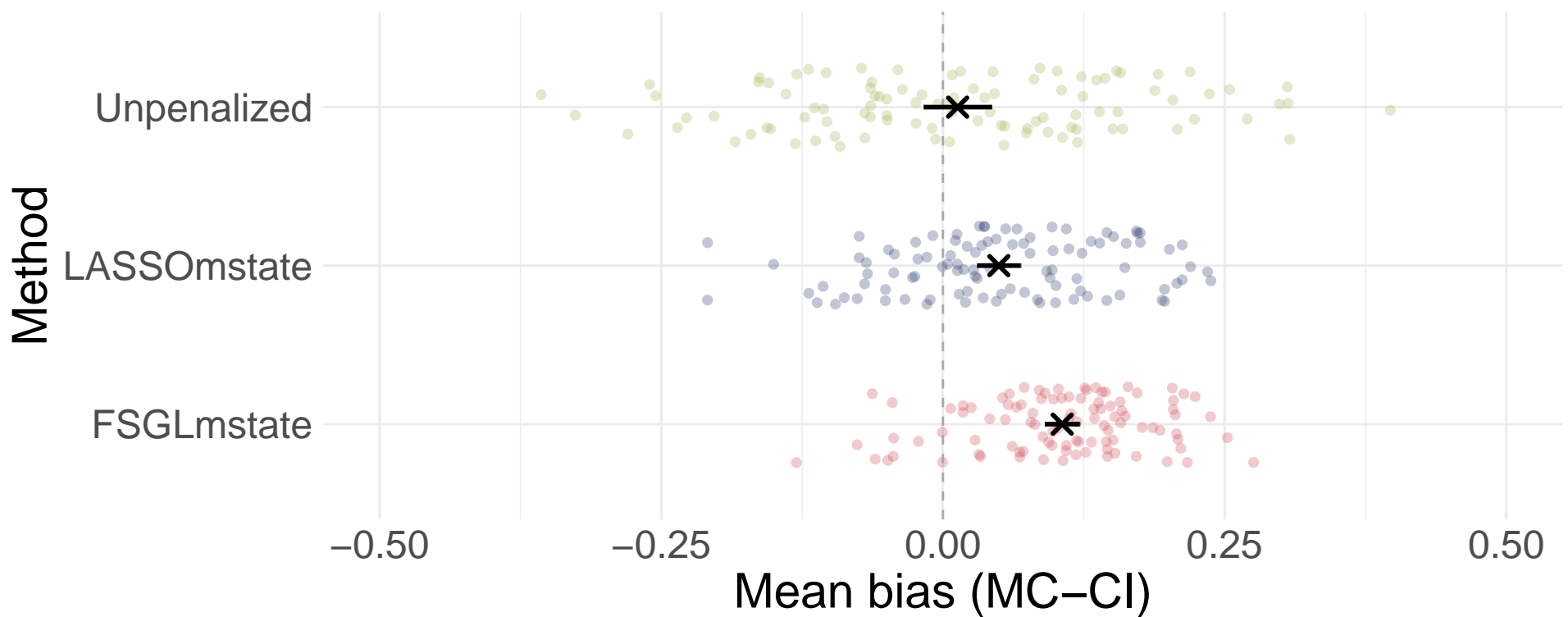

Supplement: Supplementary file 1 — Supporting Information [file BIMJ-67-e70087-s001.zip › bimj2025-sup-code/figures/Figure_A6_mean-bias_nsim100_nobs500.pdf]

Method

Unpenalized

LASSOmstate

FSGLmstate

0.00

0.25

0.50

0.75

1.00

Mean squared error (MSE)

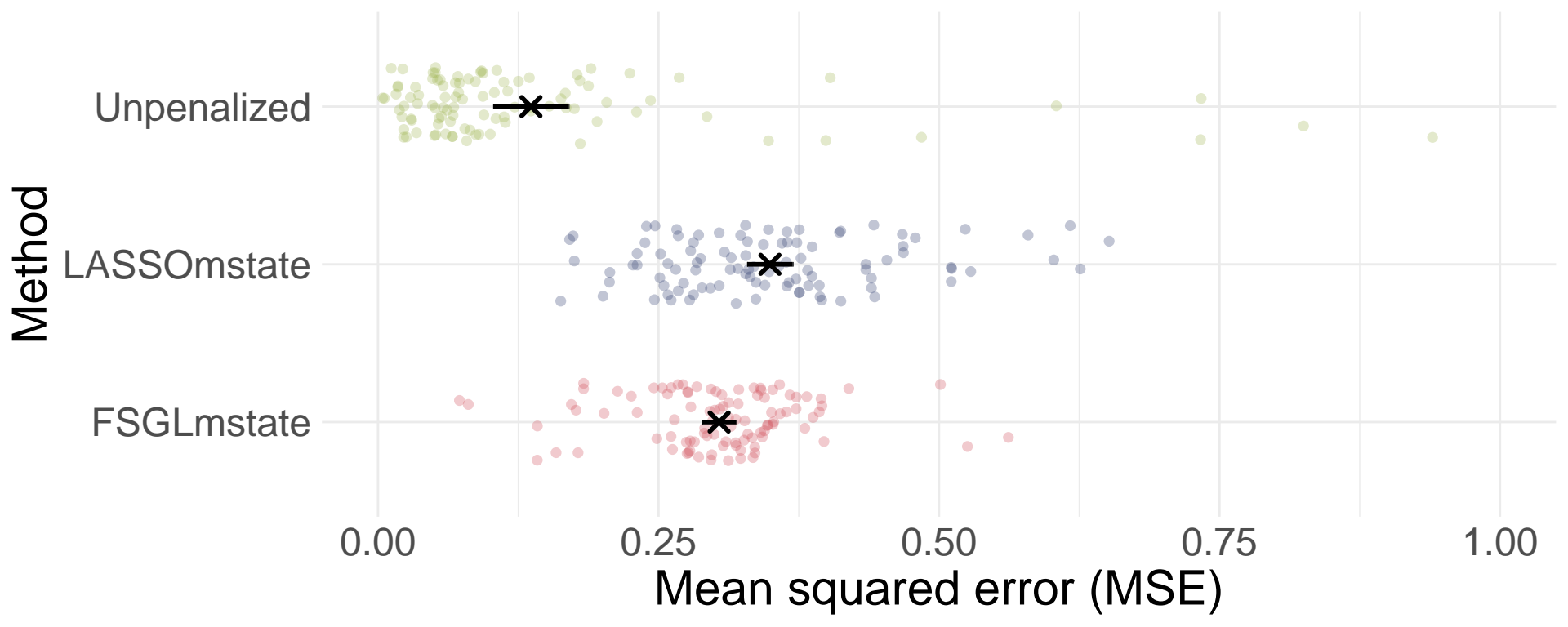

Supplement: Supplementary file 1 — Supporting Information [file BIMJ-67-e70087-s001.zip › bimj2025-sup-code/figures/Figure_A6_MSE_nsim100_nobs500.pdf]

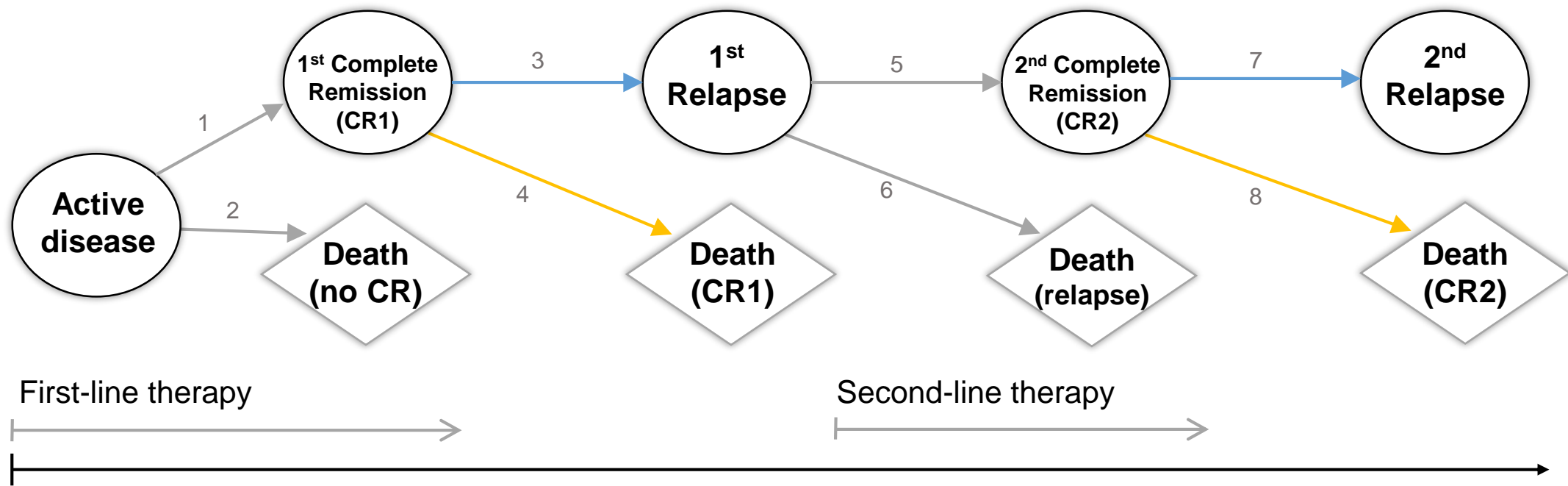

Supplement: Supplementary file 1 — Supporting Information [file BIMJ-67-e70087-s001.zip › bimj2025-sup-code/figures/Figure1_Multi-state_AML_2024.pdf]

FSGLmstate: Tuning parameter selection

Mean generalized cross-validation (GCV)

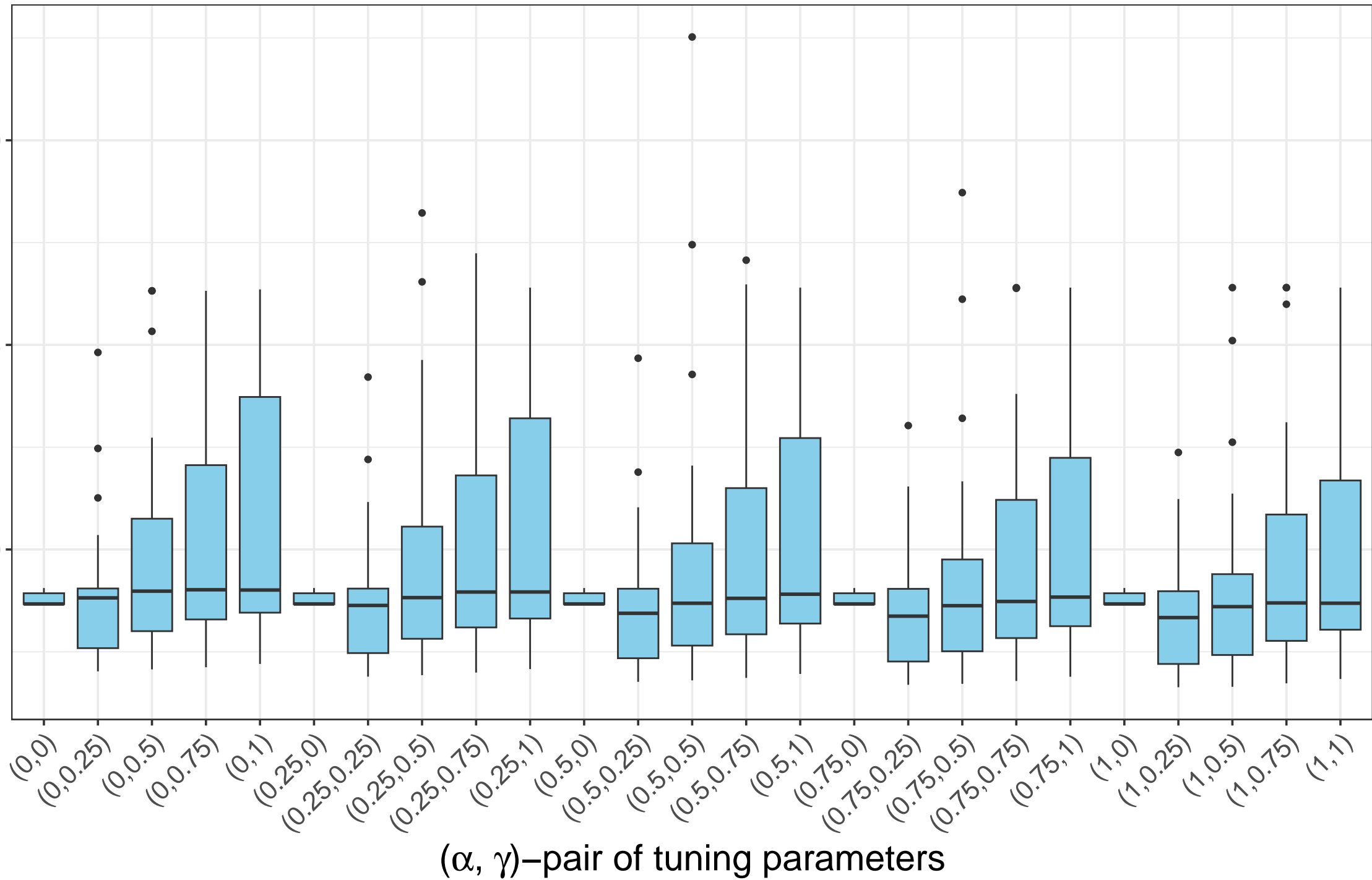

Supplement: Supplementary file 1 — Supporting Information [file BIMJ-67-e70087-s001.zip › bimj2025-sup-code/figures/Figure2_Boxplots_GCV_FSGLmstate_nobs1000_nsim225.pdf]

×  $\beta_{\text{true}}$     Method    FSGLmstate    LASSOmstate    Unpenalized

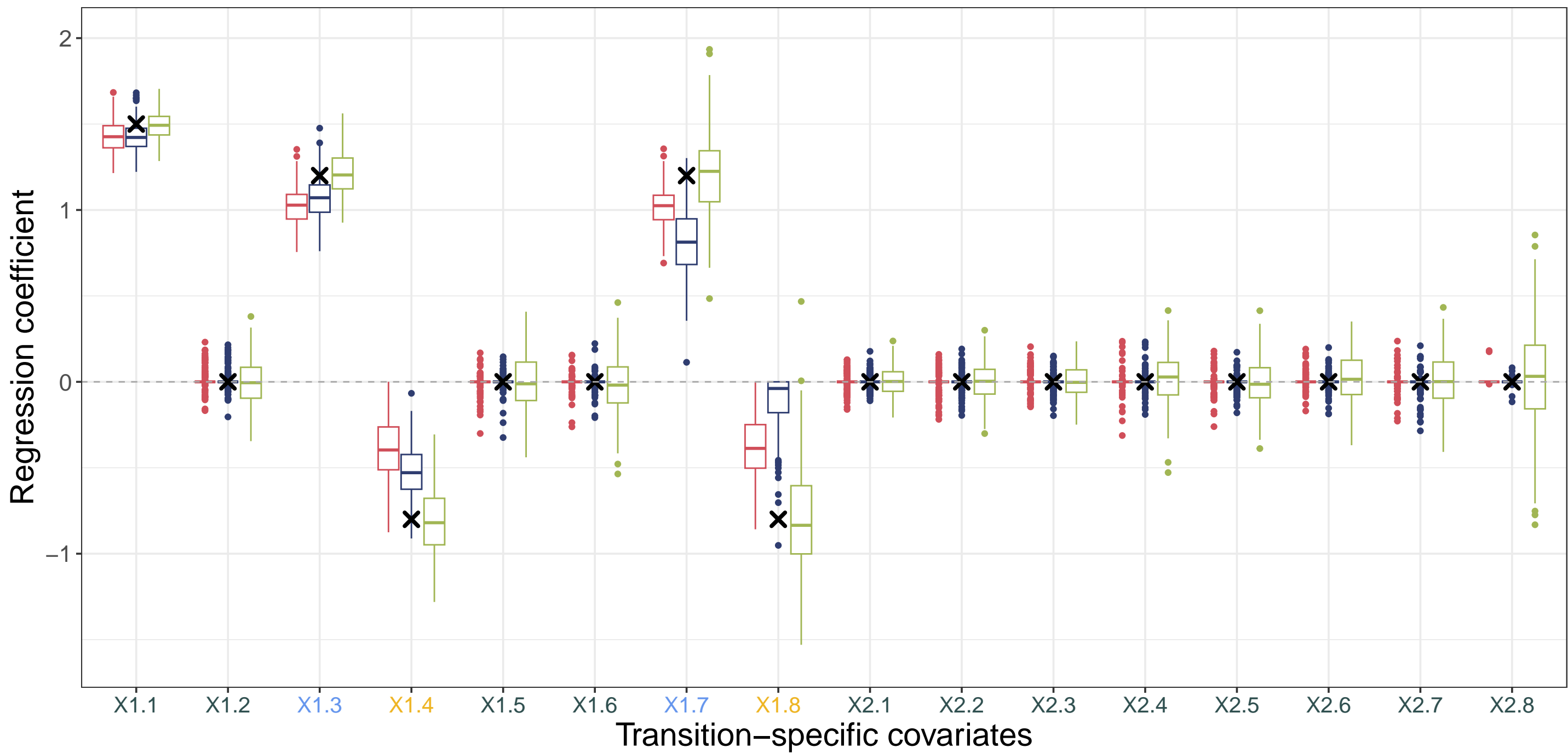

Supplement: Supplementary file 1 — Supporting Information [file BIMJ-67-e70087-s001.zip › bimj2025-sup-code/figures/Figure3_Boxplots_coef_P2_nsim225_nobs1000.pdf]

Method

LASSOmstate

FSGLmstate

0.00

0.25

0.50

0.75

1.00

False discovery rate (FDR)

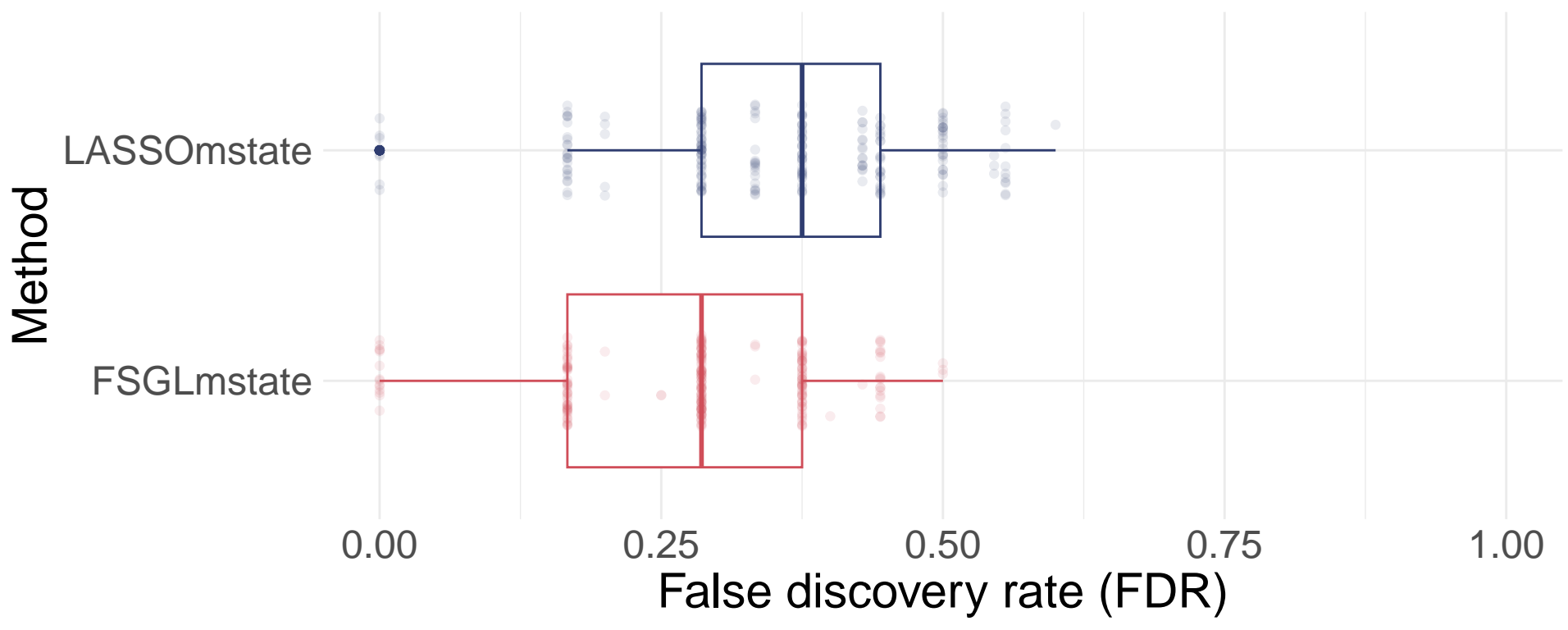

Supplement: Supplementary file 1 — Supporting Information [file BIMJ-67-e70087-s001.zip › bimj2025-sup-code/figures/Figure4_FDR_nsim225_nobs1000.pdf]

Method

LASSOmstate

FSGLmstate

0.00

0.25

0.50

0.75

1.00

True positive rate (TPR)

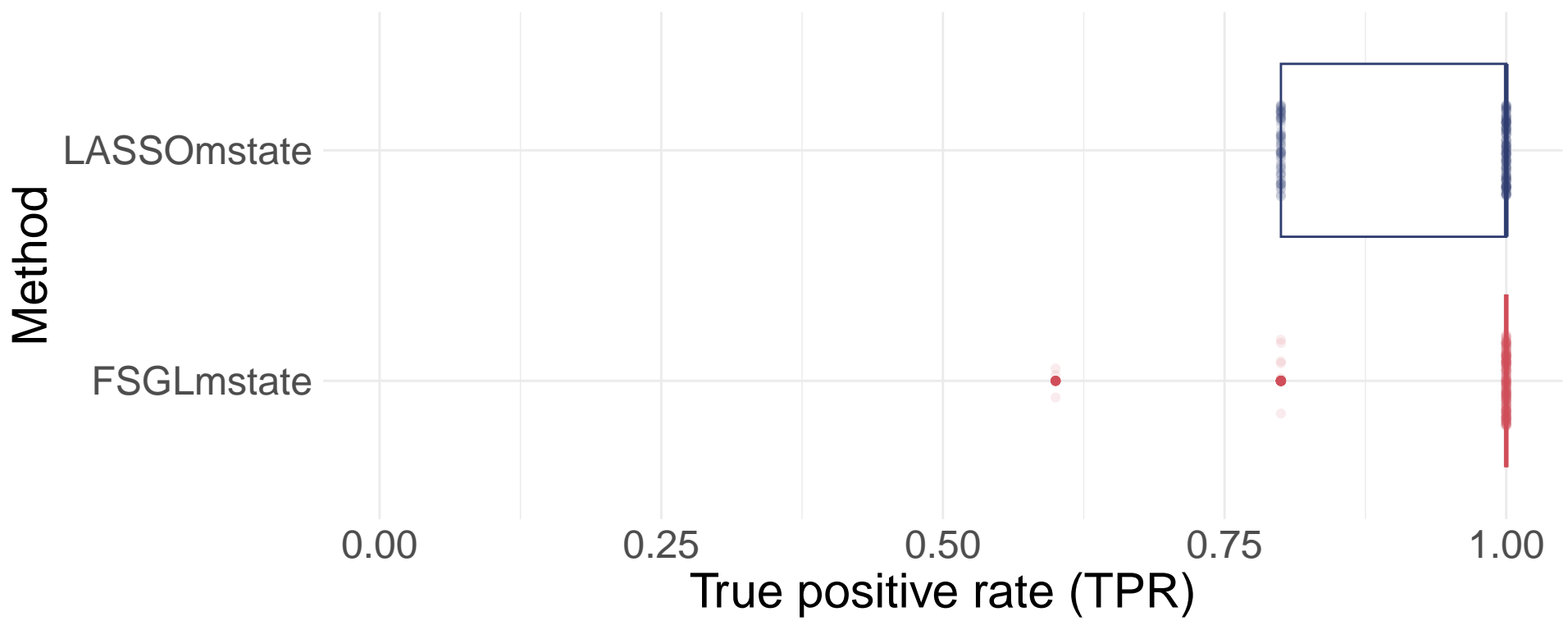

Supplement: Supplementary file 1 — Supporting Information [file BIMJ-67-e70087-s001.zip › bimj2025-sup-code/figures/Figure4_TPR_nsim225_nobs1000.pdf]

Method

Unpenalized

LASSOmstate

FSGLmstate

-0.50

-0.25

0.00

0.25

0.50

Mean bias (MC-CI)

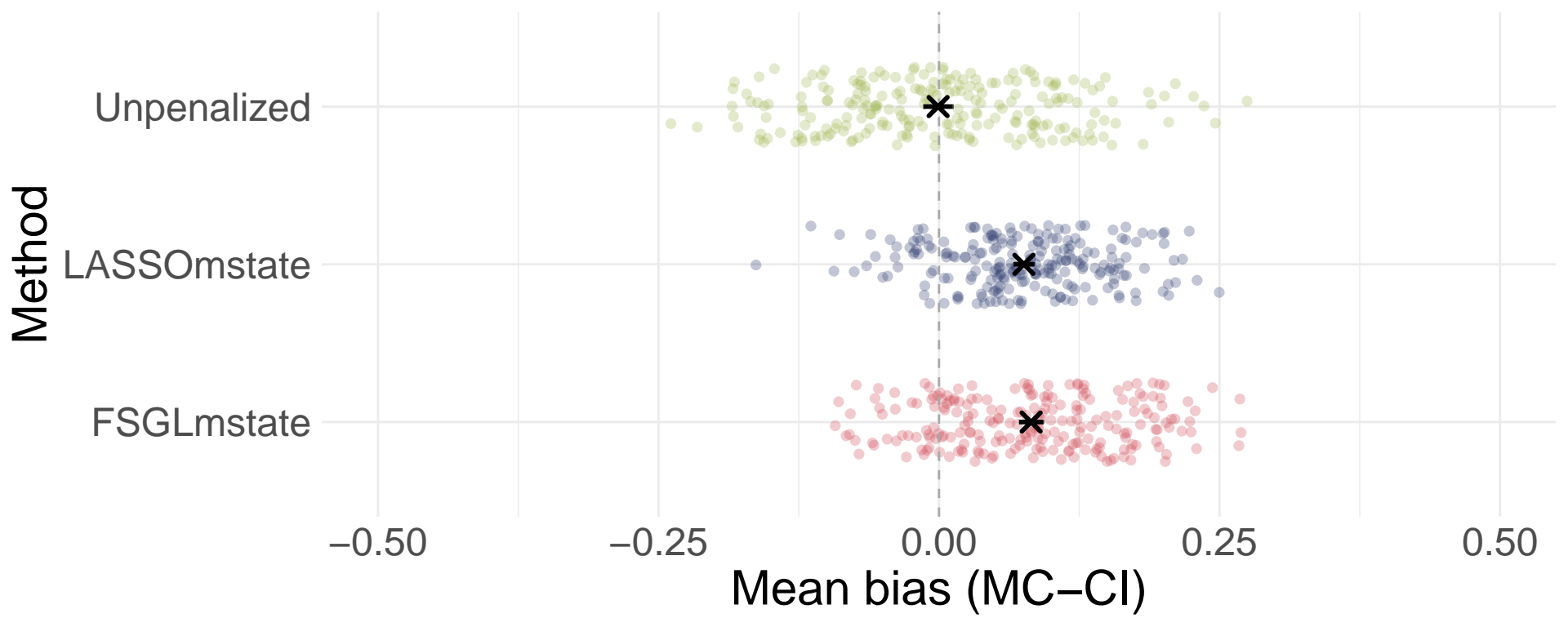

Supplement: Supplementary file 1 — Supporting Information [file BIMJ-67-e70087-s001.zip › bimj2025-sup-code/figures/Figure5_mean-bias_nsim225_nobs1000.pdf]

Method

Unpenalized

LASSOmstate

FSGLmstate

0.00

0.25

0.50

0.75

1.00

Mean squared error (MSE)

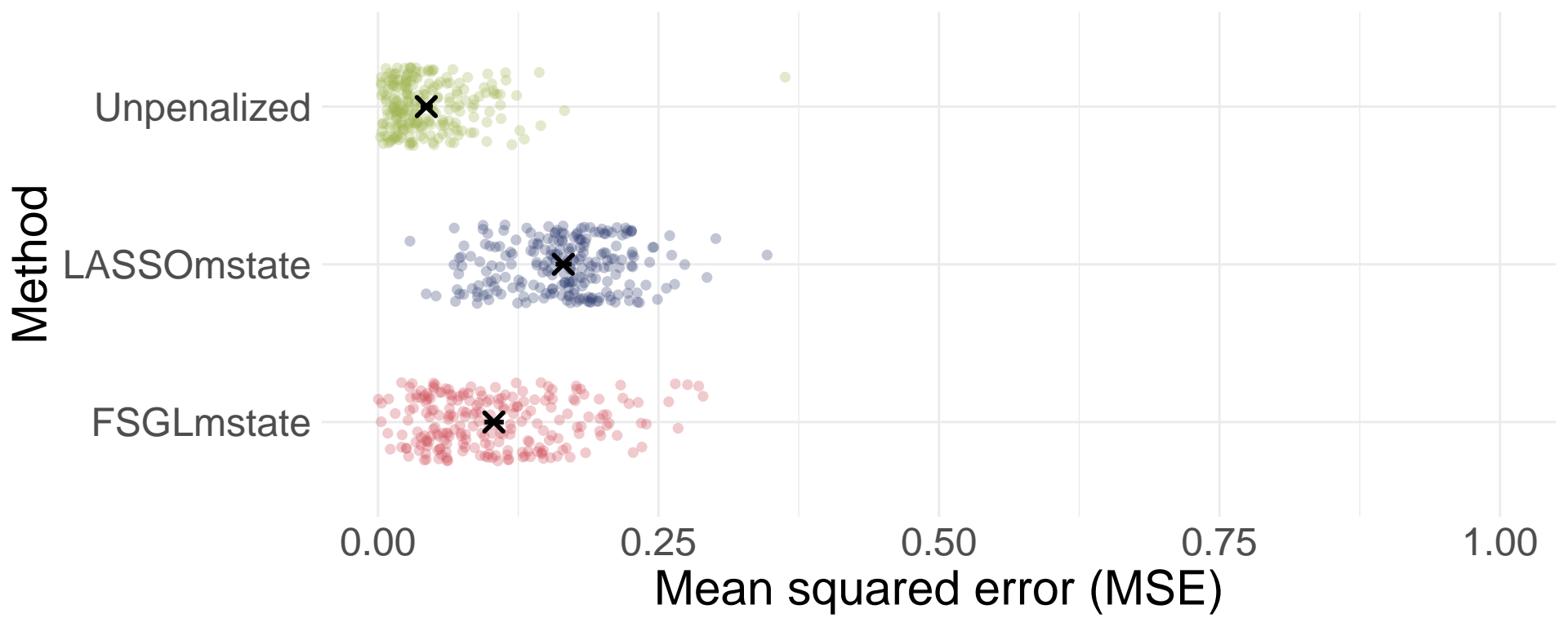

Supplement: Supplementary file 1 — Supporting Information [file BIMJ-67-e70087-s001.zip › bimj2025-sup-code/figures/Figure5_MSE_nsim225_nobs1000.pdf]

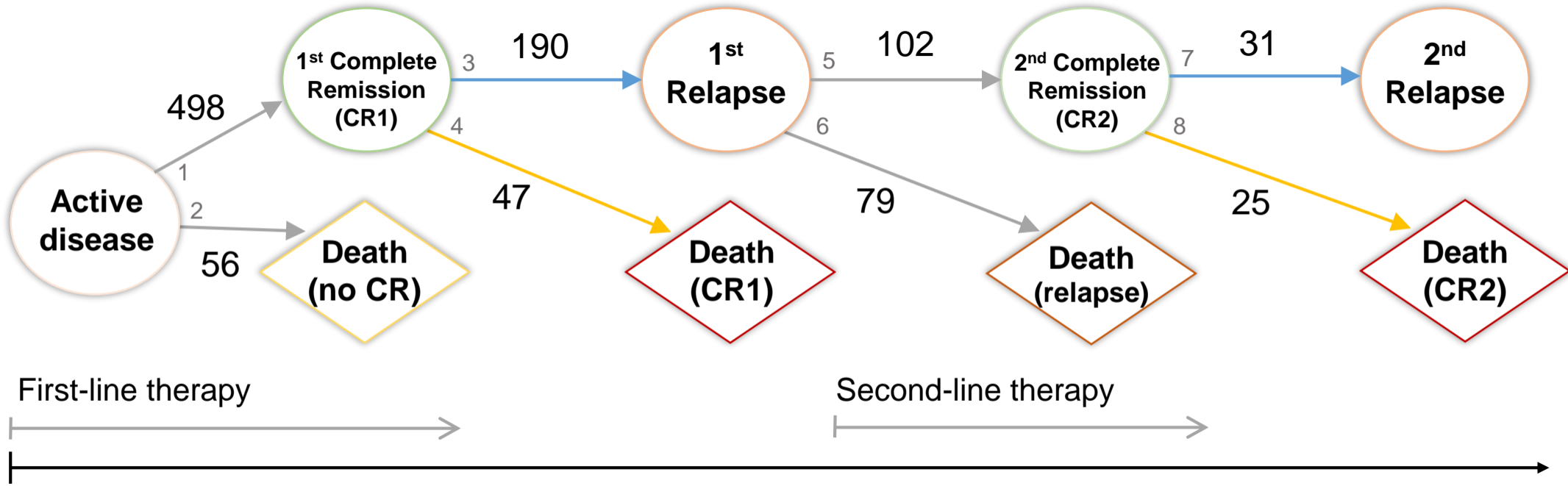

Supplement: Supplementary file 1 — Supporting Information [file BIMJ-67-e70087-s001.zip › bimj2025-sup-code/figures/Figure6_Multi-state-chart_AML_09-09.pdf]

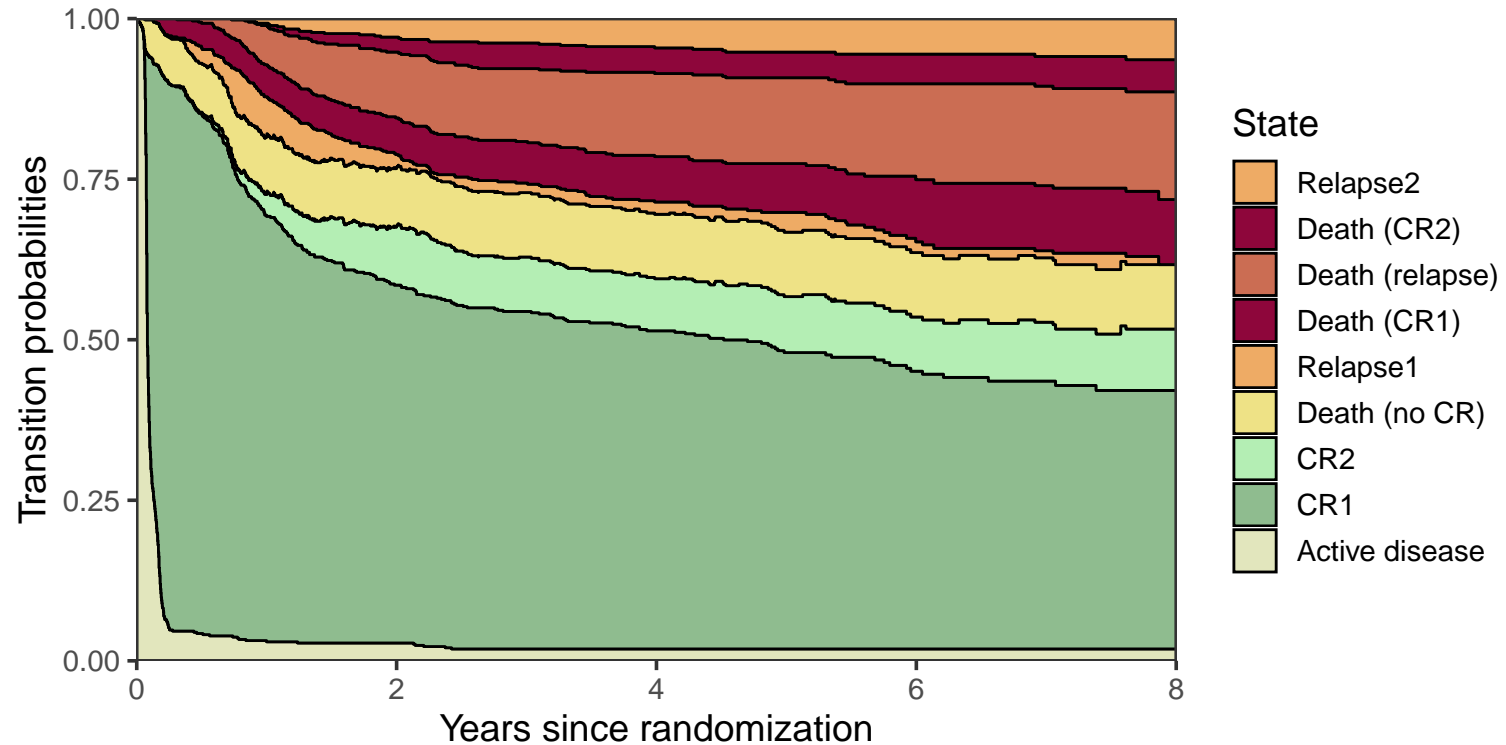

Supplement: Supplementary file 1 — Supporting Information [file BIMJ-67-e70087-s001.zip › bimj2025-sup-code/figures/Figure7_09-09_stacked-trans-probs.pdf]

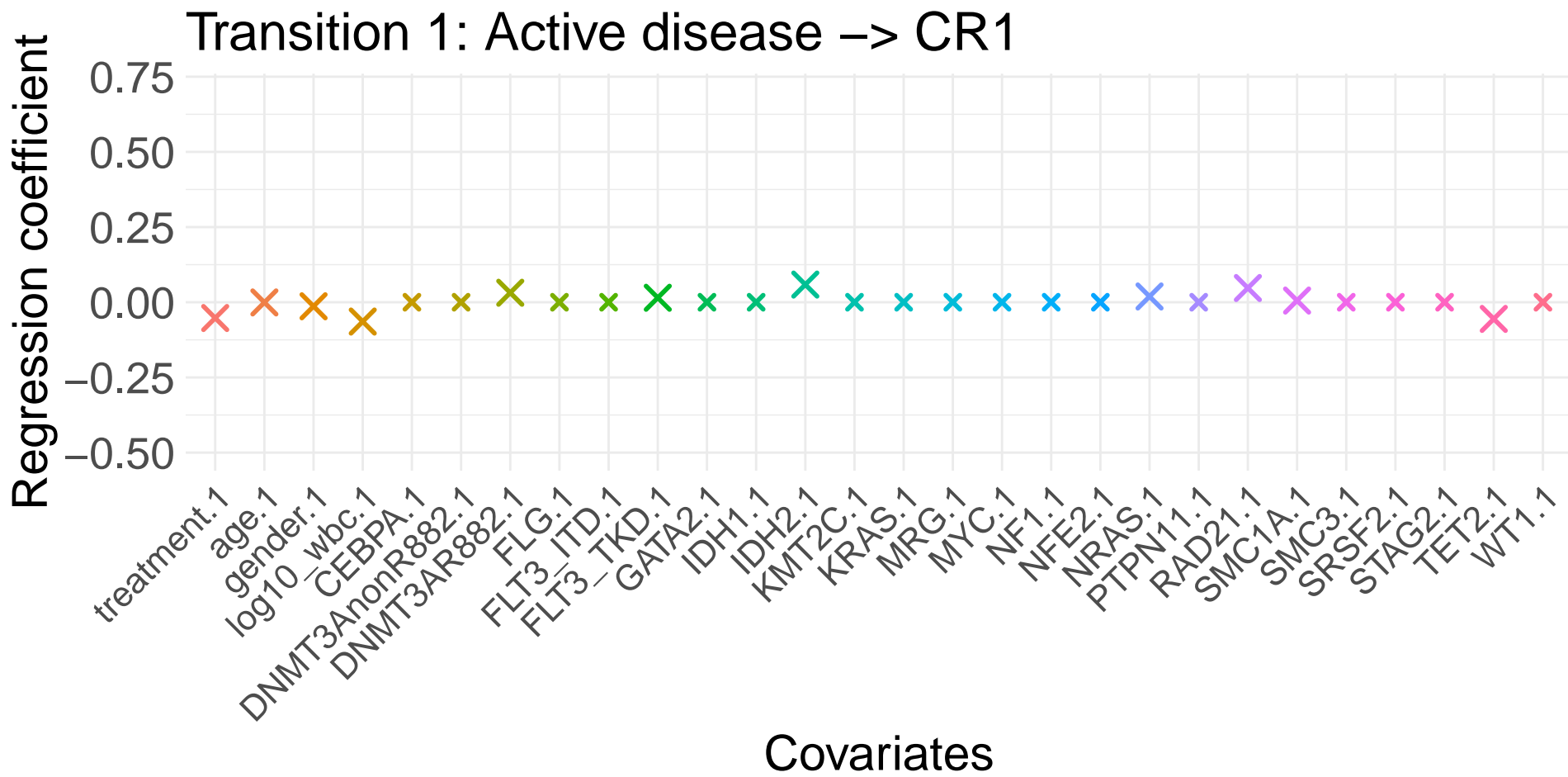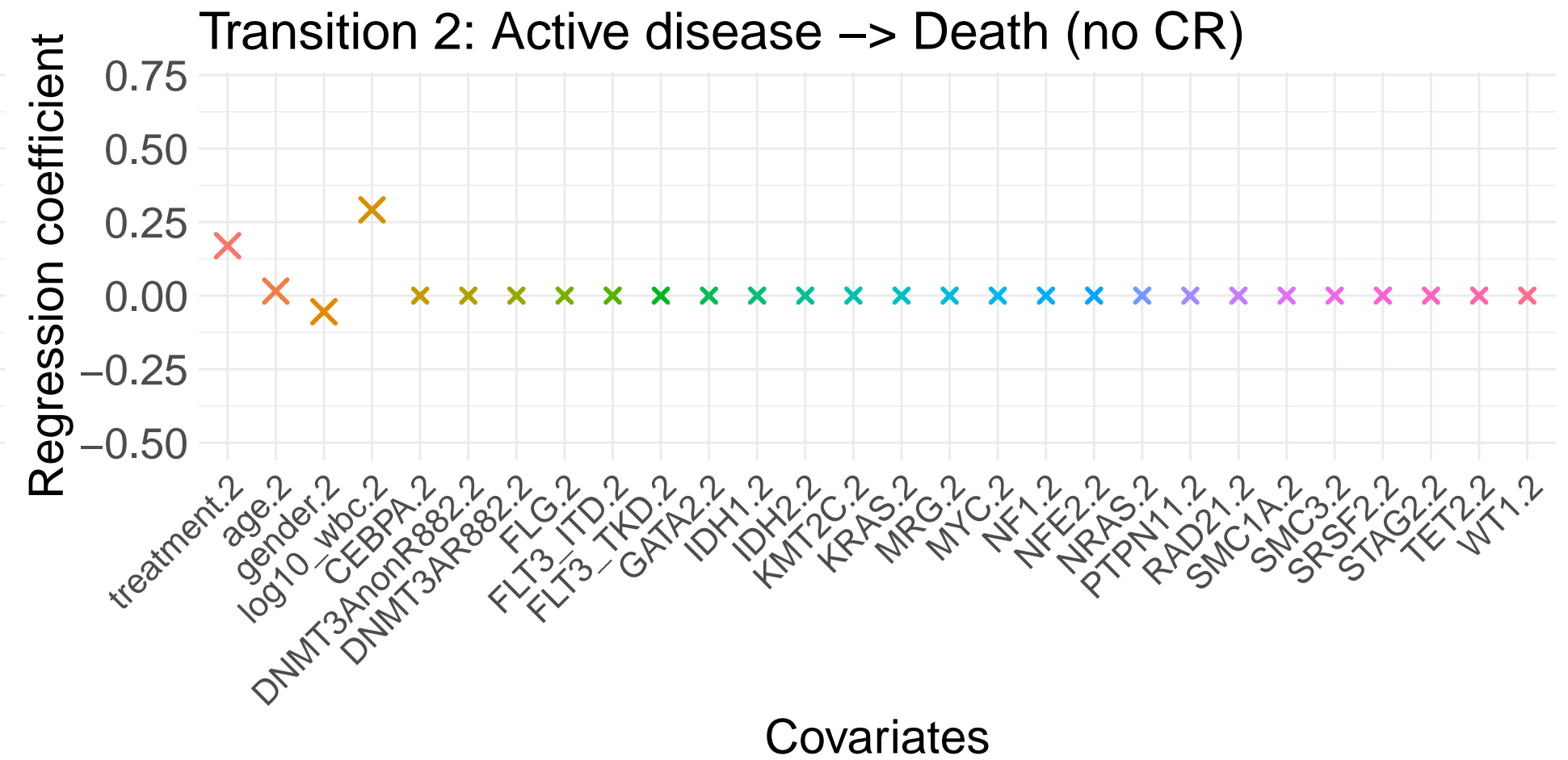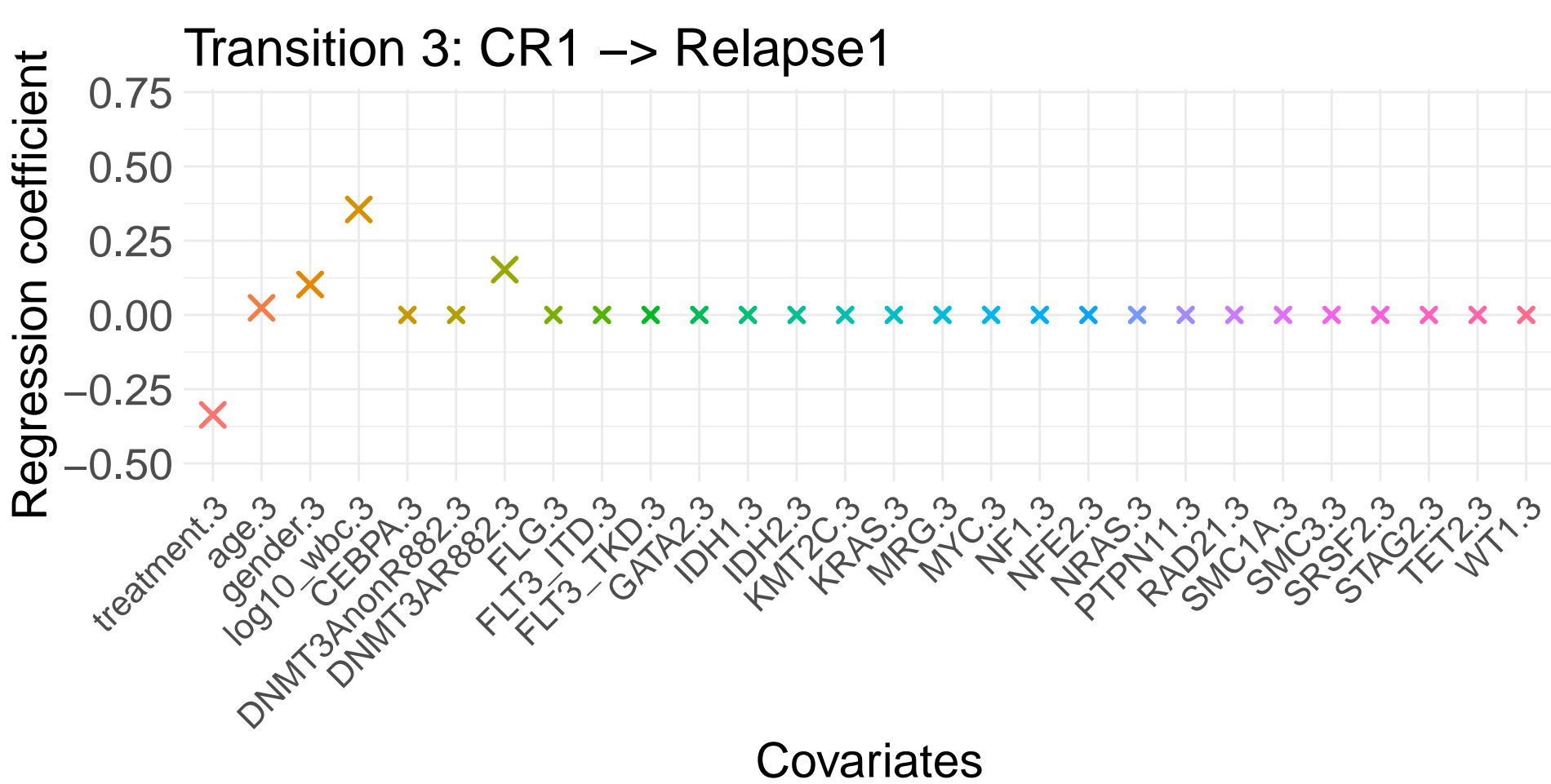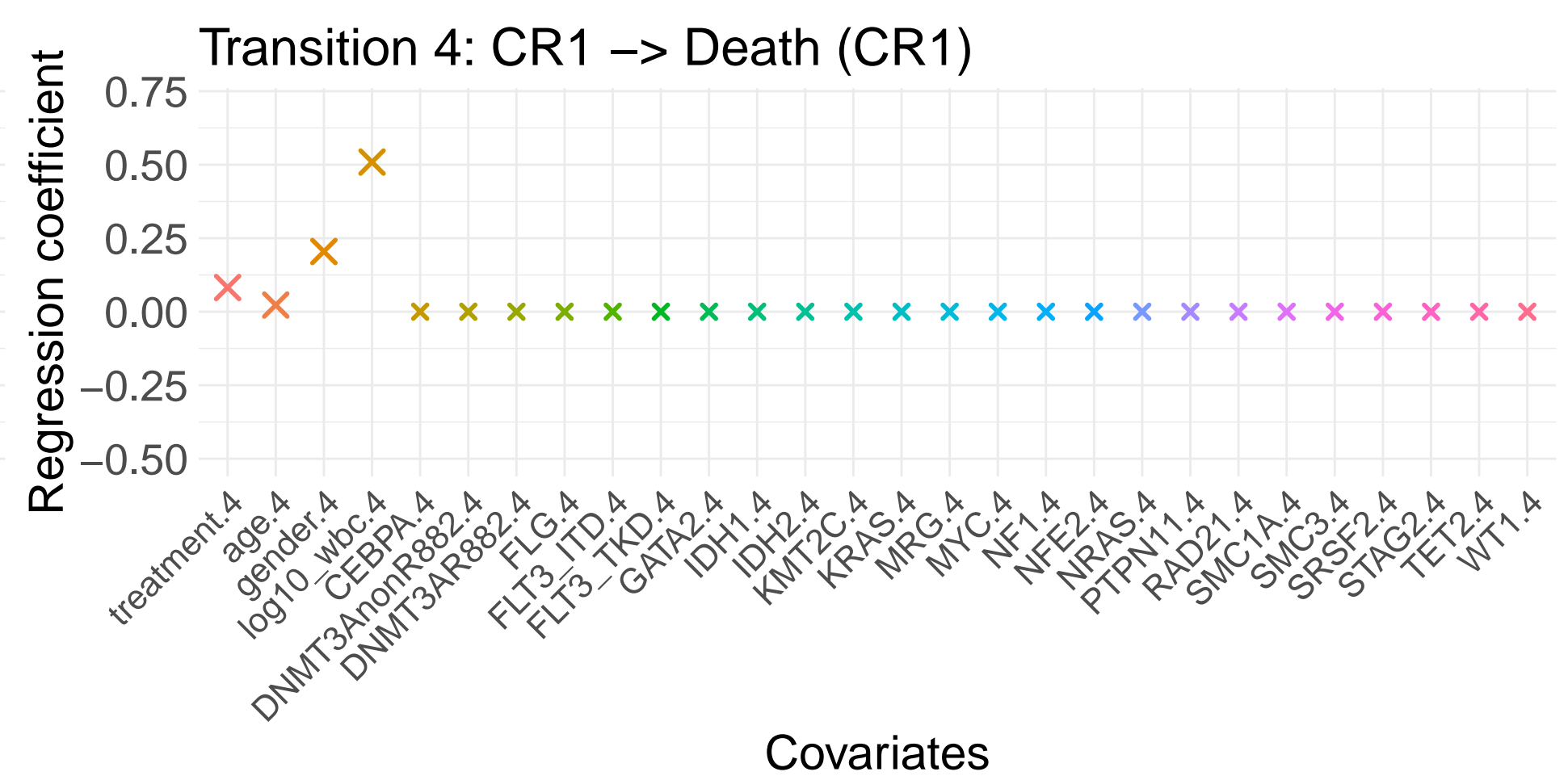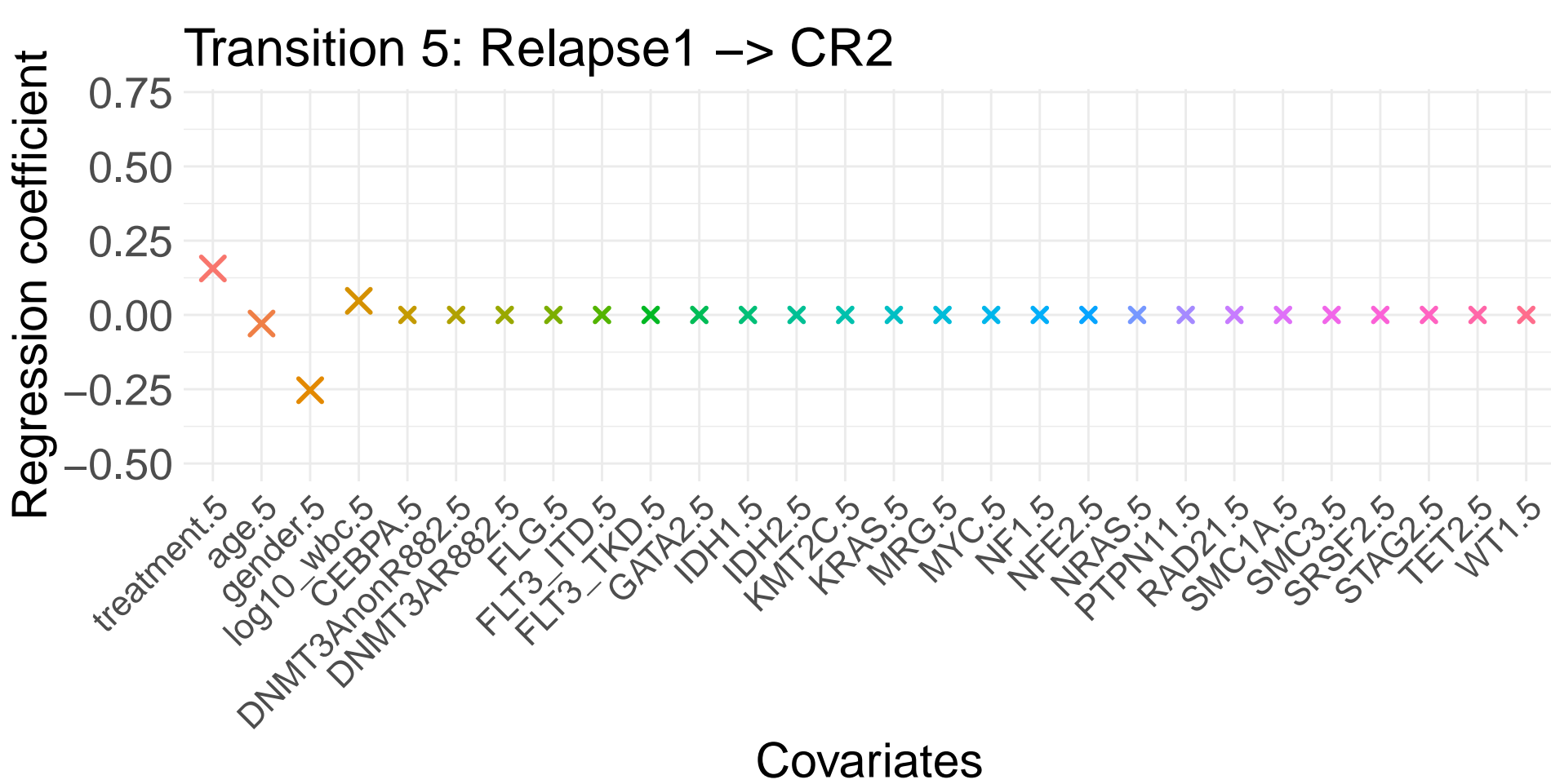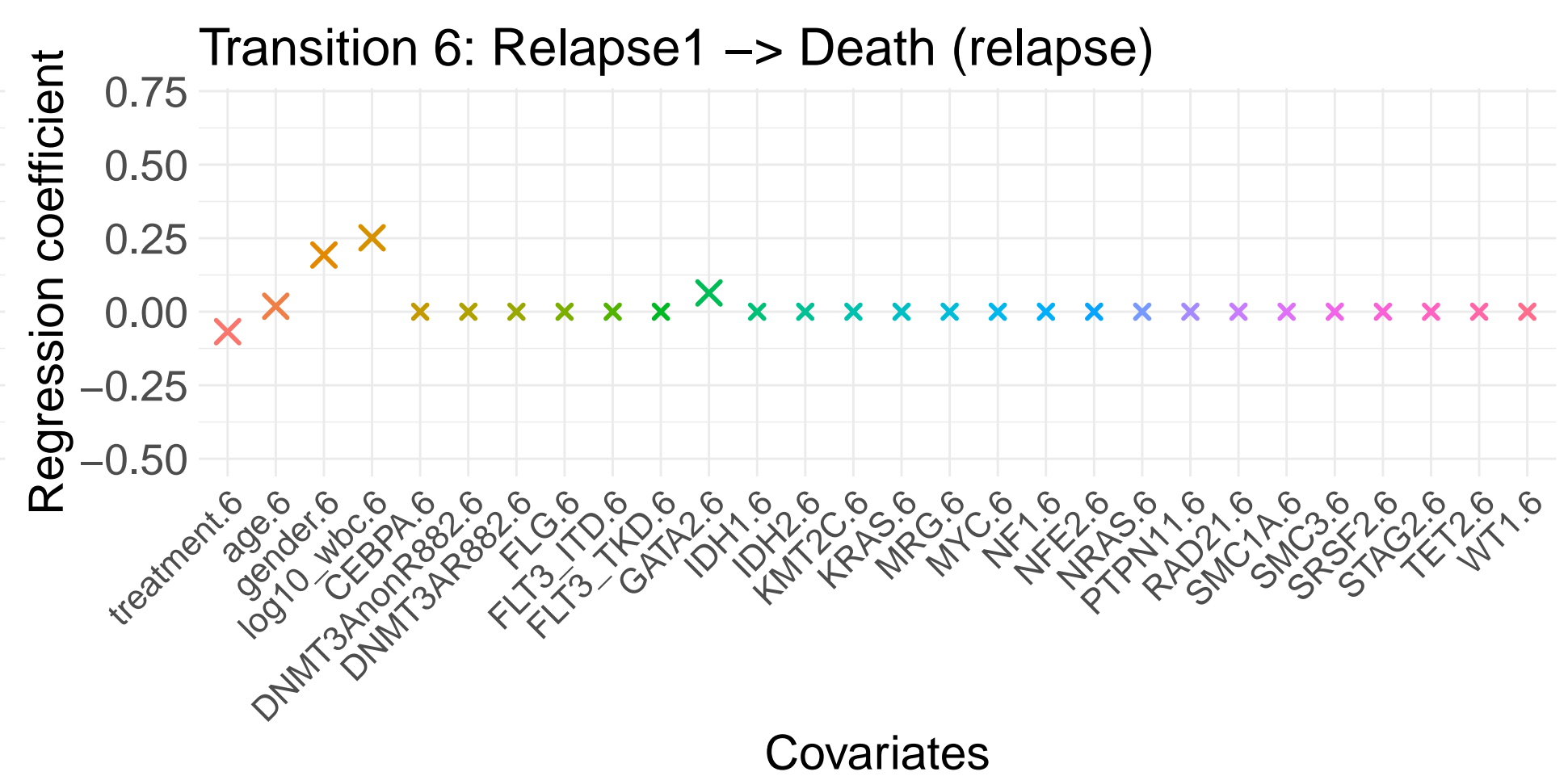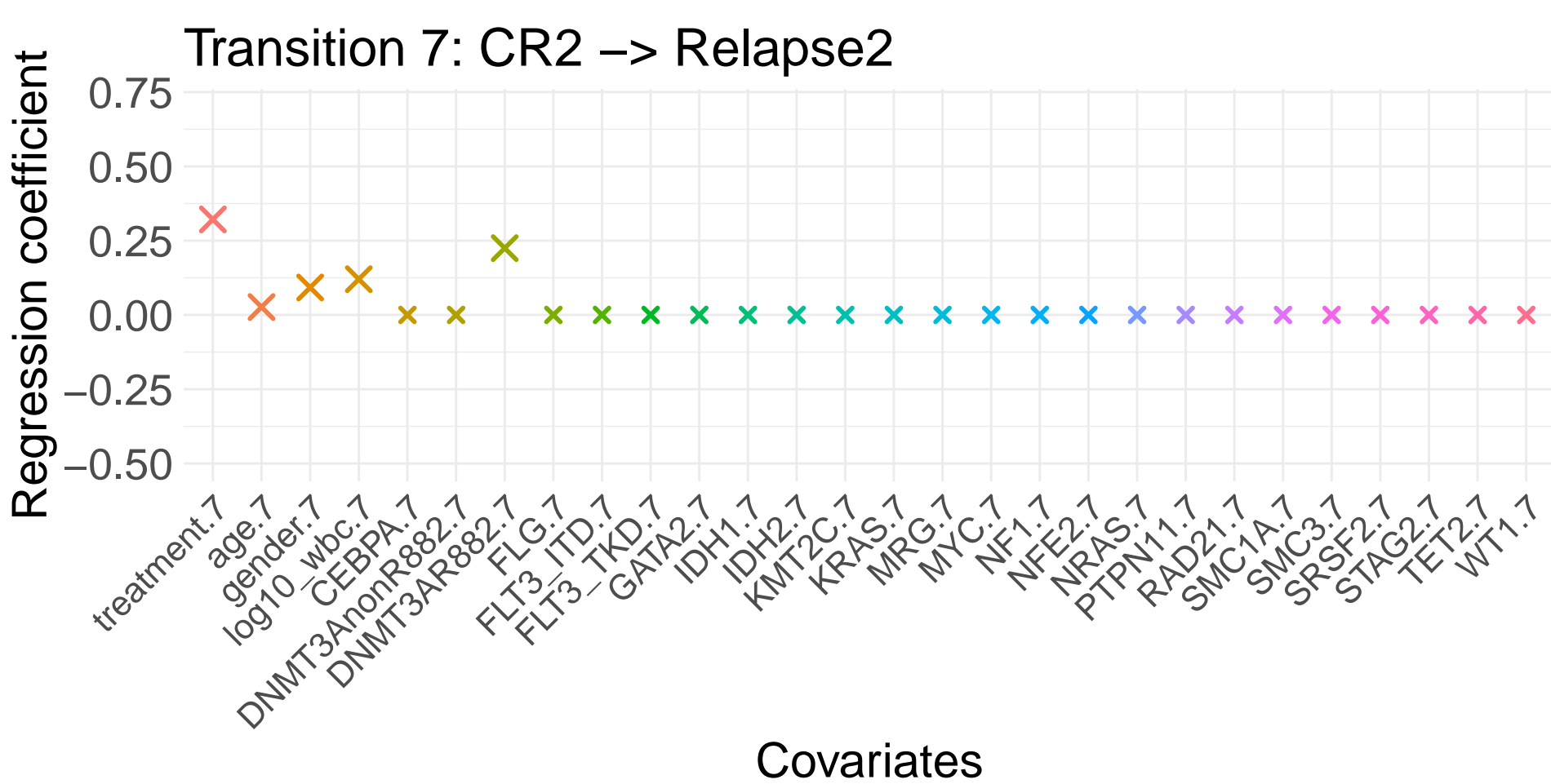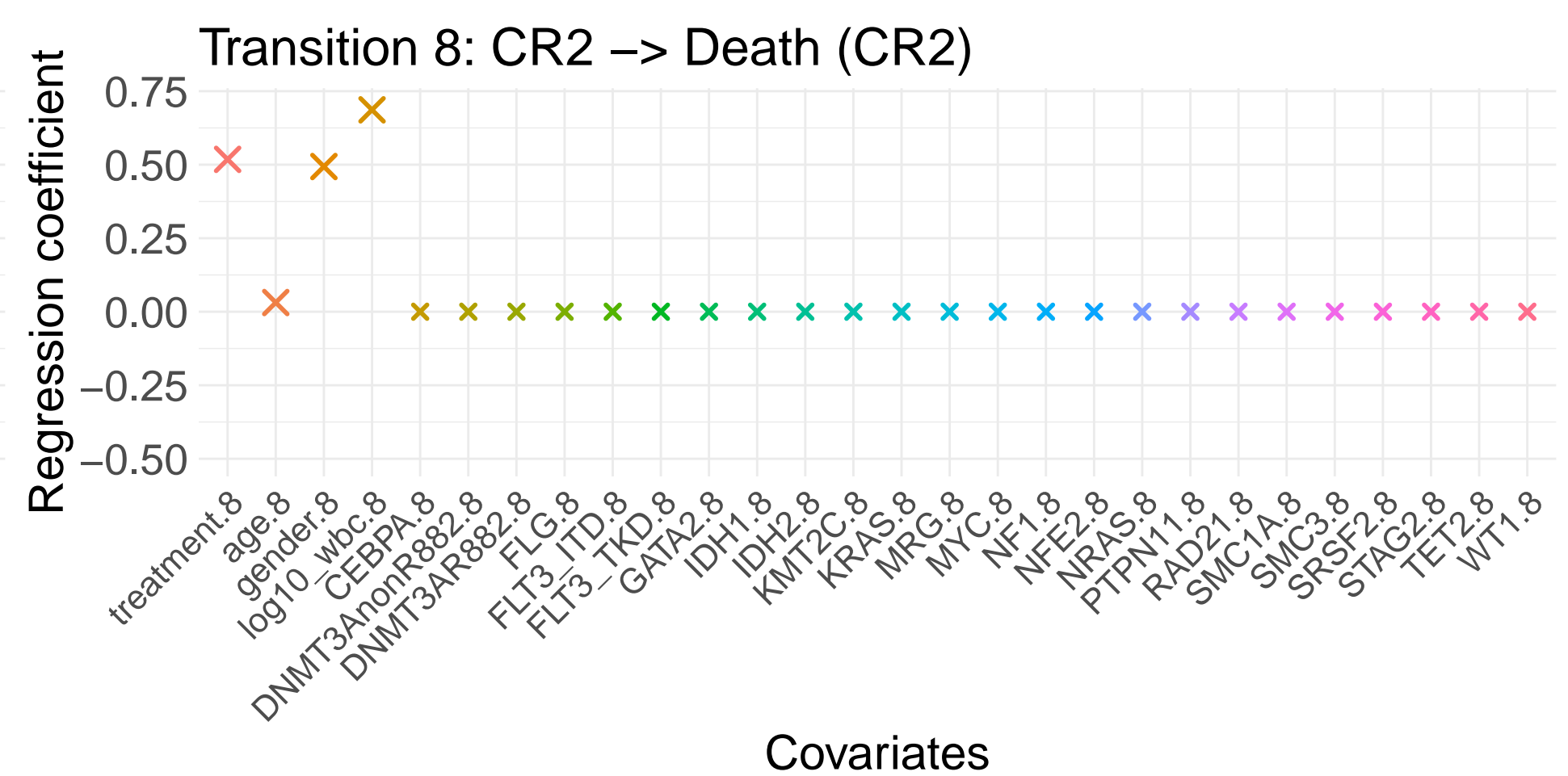

Supplement: Supplementary file 1 — Supporting Information [file BIMJ-67-e70087-s001.zip › bimj2025-sup-code/figures/Figure8_coef_09-09_FSGL_all-trans_lambda20_alpha0.75_gamma0.5.pdf]
